# Probiotics supplementation during pregnancy or infancy on multiple food allergies and gut microbiota: a systematic review and meta-analysis

**DOI:** 10.1093/nutrit/nuae024

**Published:** 2024-03-19

**Authors:** Lan Jiang, Lili Zhang, Jiayue Xia, Lei Cheng, Guoxun Chen, Jin Wang, Vijaya Raghavan

**Affiliations:** Key Laboratory of Environmental Medicine and Engineering, Ministry of Education, and Department of Nutrition and Food Hygiene, School of Public Health, Southeast University, Nanjing, China; Key Laboratory of Environmental Medicine and Engineering, Ministry of Education, and Department of Nutrition and Food Hygiene, School of Public Health, Southeast University, Nanjing, China; Key Laboratory of Environmental Medicine and Engineering, Ministry of Education, and Department of Nutrition and Food Hygiene, School of Public Health, Southeast University, Nanjing, China; Department of Otorhinolaryngology and Clinical Allergy Center, The First Affiliated Hospital, Nanjing Medical University, Nanjing, China; Department of Nutrition, University of Tennessee at Knoxville, Knoxville, TN, USA; Key Laboratory of Environmental Medicine and Engineering, Ministry of Education, and Department of Nutrition and Food Hygiene, School of Public Health, Southeast University, Nanjing, China; Department of Bioresource Engineering, Faculty of Agricultural and Environmental Sciences, McGill University, Sainte-Anne-de-Bellevue, QC, Canada

**Keywords:** children, food allergy, gut microbiota, meta-analysis, probiotics supplementation

## Abstract

**Context:**

Probiotics show promise in preventing and managing food allergies, but the impact of supplementation during pregnancy or infancy on children's allergies and gut microbiota remains unclear.

**Objective:**

This study aimed to assess the effects of maternal or infant probiotic supplementation on food allergy risk and explore the role of gut microbiota.

**Data Sources:**

A systematic search of databases (PubMed, Cochrane Library, Embase, and Medline) identified 37 relevant studies until May 20, 2023.

**Data Extraction:**

Two independent reviewers extracted data, including probiotics intervention details, gut microbiota analysis, and food allergy information.

**Data Analysis:**

Probiotics supplementation during pregnancy and infancy reduced the risk of total food allergy (relative risk [RR], 0.79; 95% CI, 0.63-0.99), cow-milk allergy (RR, 0.51; 95% CI, 0.29-0.88), and egg allergy (RR, 0.57; 95% CI, 0.39-0.84). Infancy-only supplementation lowered cow-milk allergy risk (RR, 0.69; 95% CI, 0.49-0.96), while pregnancy-only had no discernible effect. Benefits were observed with over 2 probiotic species, and a daily increase of 1.8 × 10^9^ colony-forming units during pregnancy and infancy correlated with a 4% reduction in food allergy risk. Children with food allergies had distinct gut microbiota profiles, evolving with age.

**Conclusions:**

Probiotics supplementation during pregnancy and infancy reduces food allergy risk and correlates with age-related changes in gut microbial composition in children.

**Systematic Review Registration:**

PROSPERO registration no. CRD42023425988.

## INTRODUCTION

Food allergies are on the rise and have affected millions of people worldwide.^1^^,^^2^ The global incidence of food allergies is approximately 10% in infants and 4% to 5% in older children and young adolescents.^3^ The Wayne County Health, Environment, Allergy, and Asthma Longitudinal Study (WHEALS) birth cohort study indicated that the main immunoglobulin (Ig) E–mediated food allergens in adolescents are milk, eggs, and peanuts.^4^ IgE-mediated food allergy refers to the production of food allergen–specific IgE on first exposure, and upon re-exposure to the allergen, specific IgE binds to Fc-epsilon receptor I (FcεRI) on mast cells, releasing mediators and causing acute symptoms.^5^ Food-allergic symptoms range from skin irritation to life-threatening anaphylaxis.^6^ Hence, food allergies have become a global public health concern.

Avoidance of allergic foods, food-processing desensitization, and oral immunotherapy are the major treatment strategies for food allergies. However, relevant dietary exposures, such as probiotics supplementation during pregnancy or after birth, can influence the development of food allergies.^7^ The potential role of probiotics in influencing the immune system as an innovative strategy to combat food allergies has attracted a high degree of attention. One randomized controlled trial (RCT) indicated that maternal probiotics supplementation during pregnancy can prevent egg allergy.^7^ However, another study suggested that no benefit of probiotics supplementation during infancy has been shown.^8^ Factors such as the period (during pregnancy or infancy), duration (short or long term), dose, and type of probiotics may impact the effectiveness of probiotics supplementation. However, no relevant analyses have been reported. Additionally, variations in the microbial environment may affect the risk of food allergies in offspring receiving probiotics support during pregnancy or infancy.

The development of the fetal immune system depends on the environmental exposures, ranging from the prenatal placental environment to postnatal conditions, especially during the first trimester of life.^9^^,^^10^ The gut microbiota, skin, and vasculature play an important role in the development of the immune system during early life.^9^ Gut microbiota undergo changes and maturation as individuals age. Gut microbial maturation in healthy infants undergoes a transition from *Escherichia coli* dominated, to *Bifidobacterium bifidum* dominated, eventually to Bacteroidetes dominated.^11^ However, the gut microbiota of children with food allergies differ from those of healthy children.^12^ Meanwhile, the spectrum of microbial colonization with age in children with food allergies is not clear. If the delay in maturation of the immune system is partly due to microbiota imbalance, it is then particularly important to understand age-specific microbial alterations in food-allergic infants, providing insights for early precision prevention and treatment of food allergies.

Recent reviews provided summaries of probiotics’ effects on allergic diseases; however, they did not include food allergies and did not separately address differences in supplementation during pregnancy or infancy.^13^ The objective of this meta-analysis and systematic review was to evaluate the effects of probiotics supplementation during pregnancy or infancy on overall food, cow-milk, egg, and peanut allergies in children, and to synthesize the specific microbial profiles of children during each period with food allergies.

## METHODS

### Data sources and searches

PubMed, Cochrane Library, Embase, and Medline were searched for studies related to food allergies, probiotics, and gut microbiota published until May 20, 2023. The details of the search strategy used for all databases are available in Material S1 (*please see the Supporting Information online*). The study protocol was registered prospectively in PROSPERO (https://www.crd.york.ac.uk/prospero/) with the registration number CRD42023425988. This study adhered to the Preferred Reporting Items for Systematic Reviews and Meta-Analyses (PRISMA) reporting guidelines (see Table S4 in the Supporting Information online).

### Eligibility criteria

The inclusion criteria for this meta-analysis were based on the Participants, Interventions, Comparisons, Outcomes, and Study design (PICOS) framework (Tables 1 and 2).

**Table 1:** PICOS criteria for inclusion of studies of probiotics supplementation on food allergies

Parameter	Inclusion criterion
Participants	Human studies
Interventions	Probiotics supplementation during pregnancy or infancy
Comparisons	With or without probiotics supplementation
Outcomes	Occurrence or tolerance of food allergies
Study design	Randomized controlled trial

**Table 2:** PICOS criteria for inclusion of studies of gut microbiota

Parameter	Inclusion criterion
Participants	Human studies
Interventions	Gut microbiota analysis
Comparisons	Food allergic children or healthy children
Outcomes	Gut microbial diversity
Study design	Case-control study

### Study selection and data extraction

Records obtained from the searches were downloaded and exported to EndNote X9 (Clarivate Analytics, London, UK) for de-duplication. Two independent reviewers performed 2-round screening (titles and abstracts in round 1 and full-text eligibility in round 2). Extracted data consisted of publication details, study design characteristics, probiotics intervention details, stool collection, and gut microbiota analysis. Primary outcomes of interest included the occurrence and tolerance of food allergy during pregnancy or infancy, composition of gut microbiota (alpha and beta diversity), and relative abundance at the phylum, order, family, and genus levels. Discrepancies regarding study inclusion and data interpretation were resolved by consensus through group discussions.

### Quality assessment

Randomized controlled trials of probiotics supplementation on food allergy were evaluated for risk of bias using modified versions of the Cochrane Collaboration Risk of Bias tool.^14^ The Risk of Bias tool was used to assess the randomization process, allocation concealment, blinding of participants and personnel, blinding of outcome assessment, incomplete outcome data, selective reporting bias, and other biases. The Newcastle–Ottawa Quality Assessment Scale (NOS) was used to evaluate case-control studies of gut microbiota.^15^ The NOS was assessed in 3 categories: selection, comparability, and exposure. No studies were excluded based on quality concerns. The detailed assessment is available in Tables S2 and S3 (please see the Supporting Information online).

### Statistical analysis

The main effect data were analyzed using Stata (version 11; StataCorp, College Station, TX, USA) and RevMan (version 5). Heterogeneity of the studies was quantified using *I^2^* statistics. The fixed-effects model was adopted with low heterogeneity (*I^2^* <50%). Otherwise, a random-effects model was used.

Studies on food allergies were categorized based on probiotics supplementation during pregnancy or infancy. Subgroup analysis was performed based on follow-up duration, region, and probiotics type. Sensitivity analysis was performed to investigate the influence of each study on the overall analysis. The potential nonlinear effects of probiotics dosage and follow-up duration were examined using a restricted cubic spline model.

Funnel plots and Begg’s and Egger’s tests were utilized to assess potential publication bias. A heatmap of the gut microbiota was generated using the R package pheatmap (R Foundation for Statistical Computing, Vienna, Austria). Statistical significance was set at *P <* 0.05.

## RESULTS

### Study characteristics

**Figure 1:**
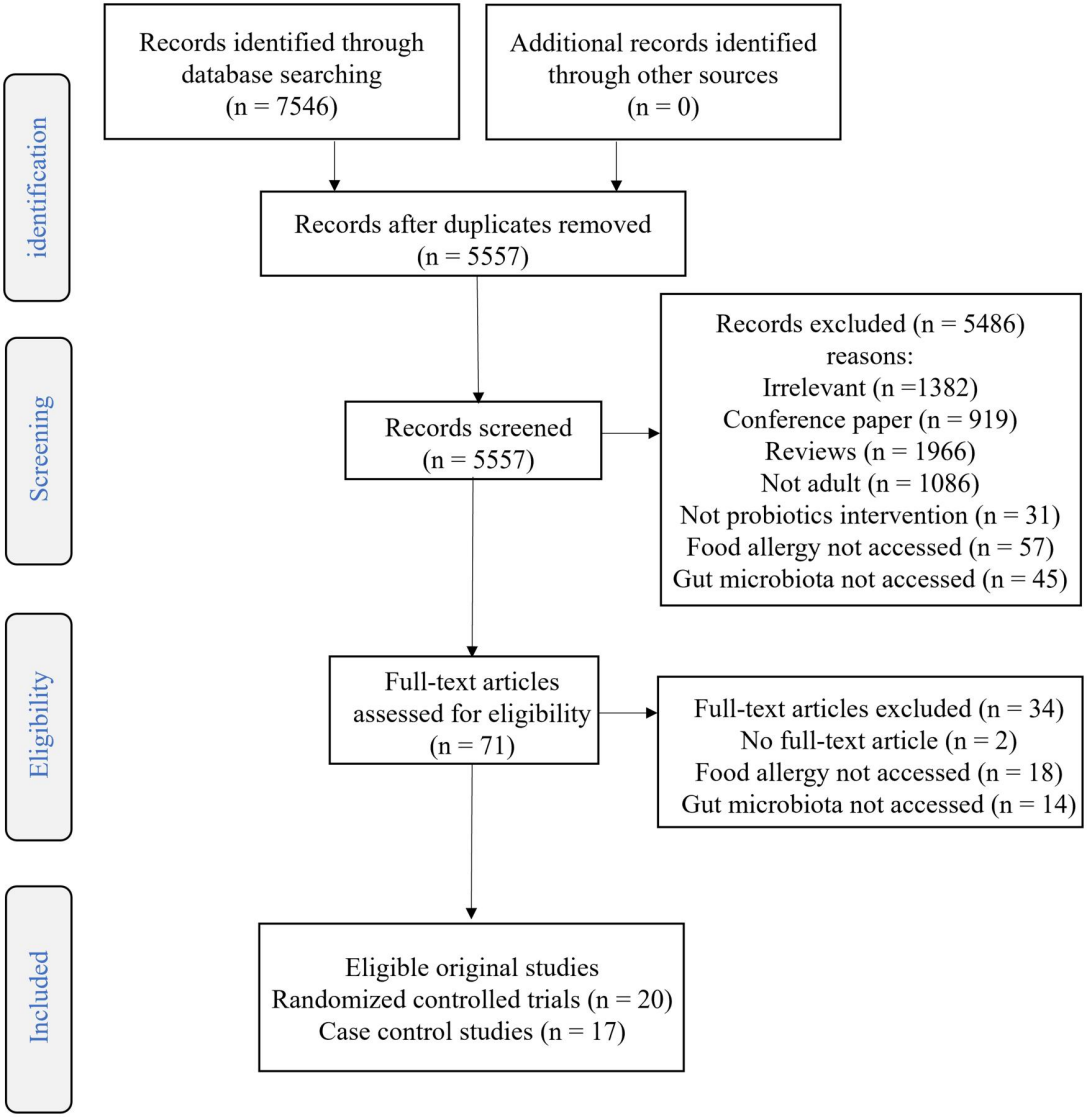
Flow diagram of the literature search process.

A total of 37 studies were included after screening, with 17 trials for probiotics supplementation on food allergy including 4 trials also for probiotics supplementation on gut microbiota, 3 trials for probiotics supplementation on tolerance towards food, and 17 case-control studies for gut microbiota (Fig. 1). The 20 RCTs captured 4597 participants in the probiotics supplementation group and 4470 participants in the control group. Seven studies^7^^,^^16–21^ for probiotics supplementation on food allergy were conducted during pregnancy and infancy, 1 study^22^ on pregnancy alone, and 9 studies^8^^,^^23–30^ on infants alone after birth; 3 studies were conducted for probiotics supplementation during infancy on tolerance towards food.^31–33^ Twelve (60.0%), 6 (30.0%), and 2 (10.0%) studies were conducted in Europe, Oceania, and Asia, respectively. The 17 trials on the risk food allergy included 15 studies on total food allergy, 10 studies on cow- milk allergy, 8 studies on egg allergy, and 6 studies on peanut allergy (Table 3).

**Table 3:** Characteristics of included studies for probiotics supplementation during pregnancy and infancy on food allergy and tolerance to foods in children

Reference	Study type	Study region	Food allergy types	Intervention period	Probiotics	Types of probiotics	Dosage
Allen (2014)^7^	RCT	UK	Egg and cow milk	Pregnant women: gestation 36 wk to delivery; infants: birth to age 6 mo (follow-up duration: 6 mo, 24 mo)	*Lactobacillus salivarius*, *Lactobacillus paracasei*, *Bifidobacterium animalis* subspecies *lactis*, and *Bifidobacterium bifidum*	4	*Lactobacillus salivarius* 6.25 × 10^9^ CFU, *Lactobacillus paracasei* 1.25 × 10^9^ CFU, *Bifidobacterium animalis* subspecies *lactis* 1.25 × 10^9^ CFU and *Bifidobacterium bifidum* 1.25 × 10^9^ CFU
Kallio (2019)^21^	RCT	Finland	All food	Pregnant women: gestation 36 wk to delivery; infants: birth to age 6 mo (follow-up duration: 13 y)	*Lactobacillus rhamnosus* GG and LC705*, Bifidobacterium breve* Bb99 and *Propionibacterium freudenreichii*	4	One capsule contains *Lactobacillus rhamnosus* GG: 5 × 10^9^ CFU, *Lactobacillus rhamnosus* LC705: 5 × 10^9^ CFU, *Bifidobacterium breve* Bb99: 2 × 10^8^ CFU, *Propionibacterium freudenreichii*: 2 × 10^9^ CFU; pregnant women: 2 capsules; infants: 1 capsule
Abrahamsson (2007)^16^	RCT	Sweden	All food, cow milk, and egg	Pregnant women: gestation 36 wk to delivery; infants: birth to age 12 mo (follow-up duration: 24 mo)	*Lactobacillus reuteri*	1	1 × 10^8^ CFU
Wickens (2008)^17^	RCT	New Zealand	All food	Pregnant women: gestation 35 wk to breastfeeding within 6 mo postpartum; infants: birth to age 24 mo (follow-up duration: 24 mo)	*Lactobacillus rhamnosus, Bifidobacterium animalis* subsp*. lactis strain*	1	*Lactobacillus rhamnosus*: 6 × 10^9^ CFU, *Bifidobacterium animalis* subsp*. lactis* strain: 9 × 10^9^ CFU
Niers (2009)^18^	RCT	Netherlands	All food	Pregnant women: 6 wk before delivery; infants: birth to age 12 mo (follow-up duration: 24 mo)	*Bifidobacterium bifidum*, *Bifidobacterium lactis*, and *Lactococcus lactis*	3	3 × 10^9^ CFU
Kim (2010)^19^	RCT	Korea	All food, cow milk, egg, and peanut	Pregnant women: 8 wk before delivery to 3 mo after delivery; infants: birth to age 4–6 months (follow-up duration: 12 mo)	*Bifidobacterium bifidum*, *Bifidobacterium lactis*, and *Lactobacillus acidophilus*	3	*Bifidobacterium bifidum*: 1.6 × 10^9^ CFU; *Bifidobacterium lactis*: 1.6 × 10^9^ CFU; *Lactobacillus acidophilus*: 1.6 × 10^9^ CFU
Boyle (2011)^22^	RCT	Australia	All food, cow milk, egg, and peanut	Pregnant women: gestation 36 wk to delivery (follow-up duration: 12 mo)	*Lactobacillus rhamnosus* GG	1	1.8 × 10^10^ CFU
Kalliomäki (2003)^20^	RCT	Finland	Cow milk and peanut	Pregnant women: 4 wk before delivery to 6 mo after delivery (the mother or the infant consumed the preparation)	*Lactobacillus rhamnosus* GG	2	1 × 10^10^ CFU
Plummer (2019)^23^	RCT	Australia	All food	Infants: very preterm infants from soon after birth until discharge from hospital or term corrected age	*Bifidobacterium infanti*, *Streptococcus thermophilus*, and *Bifidobacterium lactis*	3	*Bifidobacterium infantis*: 3 × 10^8^; *Streptococcus thermophilus*: 3.5 × 10^8^; *Bifidobacterium lactis*: 3.5 × 10^8^ per 1.5 g base powder
Morisset (2008)^24^	RCT	France	All food and cow milk	Infants: birth to age 12 mo (follow-up duration: 4, 12, and 24 mo)	Heat-killed *Bifidobacterium breve* C50 and *Streptococcus thermophilus* 065	2	*Bifidobacterium breve* C50: 4.2 × 10^9^ bacteria per 100 g of powder formula; *Streptococcus thermophilus* 065: 3.84 × 10^7^ bacteria per 100 g of powder formula
Taylor (2007)^25^	RCT	Australia	All food, cow milk, egg, and peanut	Infants: birth to age 6 mo (follow-up duration: 12 mo)	*Lactobacillus acidophilus*	1	3 × 10^9^ CFU
Prescott (2008)^26^	RCT	Australia	All food, cow milk, egg, and peanut	Infants: birth to age 6 mo (follow-up duration: 30 mo)	*Lactobacillus acidophilus*	1	3 × 10^9^ CFU
Jensen (2012)^8^	RCT	Australia	All food, cow milk, egg, and peanut	Infants: birth to age 6 mo (follow-up duration: 5 y)	*Lactobacillus acidophilus*	1	3 × 10^9^ CFU
West (2013)^27^	RCT	Sweden	All food	Infants: 4 to 13 mo (follow-up duration: 8–9 y)	*Lactobacillus paracasei* ssp*. paracasei* F19	1	1 × 10^8^ CFU
Soh (2009)^28^	RCT	Singapore	All food and egg	Infants: birth to age 6 mo (follow-up duration: 12 mo)	*Bifidobacterium longum* and *Lactobacillus rhamnosus*	2	At least 2.8 × 10^8^ CFU
Rautava (2006)^29^	RCT	Finland	All food and cow milk	Infants: 2 to 12 mo (follow-up duration: 12 mo)	*Lactobacillus rhamnosus* and *Bifidobacterium lactis* Bb-12	2	1 × 10^10^ CFU
Lau (2012)^30^	RCT	Germany	All food	Infants: 5 to 31 wk (follow-up duration: 36 mo)	Heat-killed nonpathogenic gram-negative *Esherichia coli Symbio* and nonpathogenic gram-positive *E. faecalis Symbio*	2	3.15–9.45 × 10^7^ bacteria
Hol (2008)^31^	RCT	Netherlands	Cow milk	Infants: 6 and 12 mo after initial cow-milk allergy diagnosis	*Lactobacillus casei* CRL431 and *Bifidobacterium lactis* Bb-12	2	1 × 10^7^ CFU/g formula
Berni Canani (2012)^32^	RCT	Italy	Cow milk	Infants: 6 and 12 mo after initial cow-milk allergy diagnosis	*Lactobacillus rhamnosus* GG	1	6.5 × 10^7^ CFU/g formula
Berni Canani (2013)^33^	RCT	Italy	Cow milk	Infants: 12 mo after initial cow-milk allergy diagnosis	*Lactobacillus rhamnosus* GG	1	Not indicated

*Abbreviations:* CFU, colony-forming unit; RCT, randomized controlled trial.

Seventeen studies on gut microbiota included 706 patients with food allergies and 913 healthy controls.^34–50^ They included 7 studies in Asia, 7 in America, 2 in Europe, and 1 in Oceania. No difference in sex was found between the food allergy and control groups. The ages of the participants ranged from 0 to 18 years (Table 4). The collection, preservation, and extraction of intestinal samples and the methods of β-diversity analysis are shown in Table S1 (please see the Supporting Information online).

**Table 4:** Characteristics of included studies for perturbations of gut microbiota composition in children with food allergy

Reference	Study type	Study region	No. of patients	Age	Female, %	Food allergy types
Bao (2021)^34^	Case control	USA	34	39.4 ± 4.1 y (M ± SEM)	64.7%	Food allergy
Bunyavanich (2016)^35^	Case control	USA	226	3.6–16.9 mo (range)	51.7%	Milk
Schink (2018)^36^	Case-control	Germany	31	38.1 ± 15.1 y (M ± SD)	83.9%	—
Du (2020)^37^	Case-control	China	50	33.0 ± 11.7 y (M ± SD)	48.0%	Wheat-dependent, exercise-induced anaphylaxis
Fazlollahi (2018)^38^	Case control	USA	141	9.5 (7.1–12.3) mo (IQR)	32.6%	Egg
Goldberg (2020)^39^	Case-control	USA	291	Allergic: 77 (63.0–114.5) mo Control: 78 (48.0–125.3) mo (M, IQR)	40.9%	Milk, peanut, sesame, tree nuts
Kourosh (2018)^40^	Case-control	USA	42	0–18 y (range)	52.4%	Peanut/tree nuts, fish, milk, egg, sesame, soy, wheat, chick pea, lentil, avocado, green pea
Lee (2021)^41^	Case-control	Australia	60	1–7 y (range)	60.7%	Nuts, egg, and mixed allergies
Savage (2018)^44^	Case-control	USA	216	Allergic: 153 (115–208) d Control: 142 (78–206) d (M, range)	49.1%	Milk, egg, peanut, soy, wheat, walnut
Dong (2018)^45^	Case-control	China	120	2.9 ± 1 mo (M ± SD)	46.7%	Milk
Ling (2014)^46^	Case-control	China	79	5.4 ± 2.1 mo (M ± SD)	50.6%	Milk, eggs, wheat, nut, peanuts, fish, shrimp, and soybeans
Łoś-Rycharska (2021)^47^	Case-control	Poland	44	15.1 ± 6.6 wk (M ± SD)	40.9%	Milk
Yamagishi (2021)^48^	Case-control	Japan	40	Allergic: 3.1 (1.5–5.5) ys Control: 4.0 (2.9–6.1) y (M, IQR)	NA	Egg
Azad (2015)^49^	Case-control	Canada	166	3 mo or 1 y	48.8%	—
Tanaka (2017)^50^	Case-control	Japan	41	1 y	34.1%	Egg, milk, wheat, soybean
Dong (2018)^42^	Case-control	China	14	5–8 y (range)	Sex-matched	Milk
Guo (2016)^43^	Case-control	China	24	5–8 y (range)	50.0%	Milk

*Abbreviations:* IQR, interquartile range; M, mean; NA, not applicable; SD, standard deviation; SEM, standard error of mean.

Sensitivity analyses did not reveal any change in the main effects of probiotics on food allergies and gut microbiota diversity. Funnel plots showed a slight publication bias, but not all effects were significant according to Egger's parametric or Begg's nonparametric distribution tests.

### Probiotics supplementation on multiple food allergies

**Figure 2:**
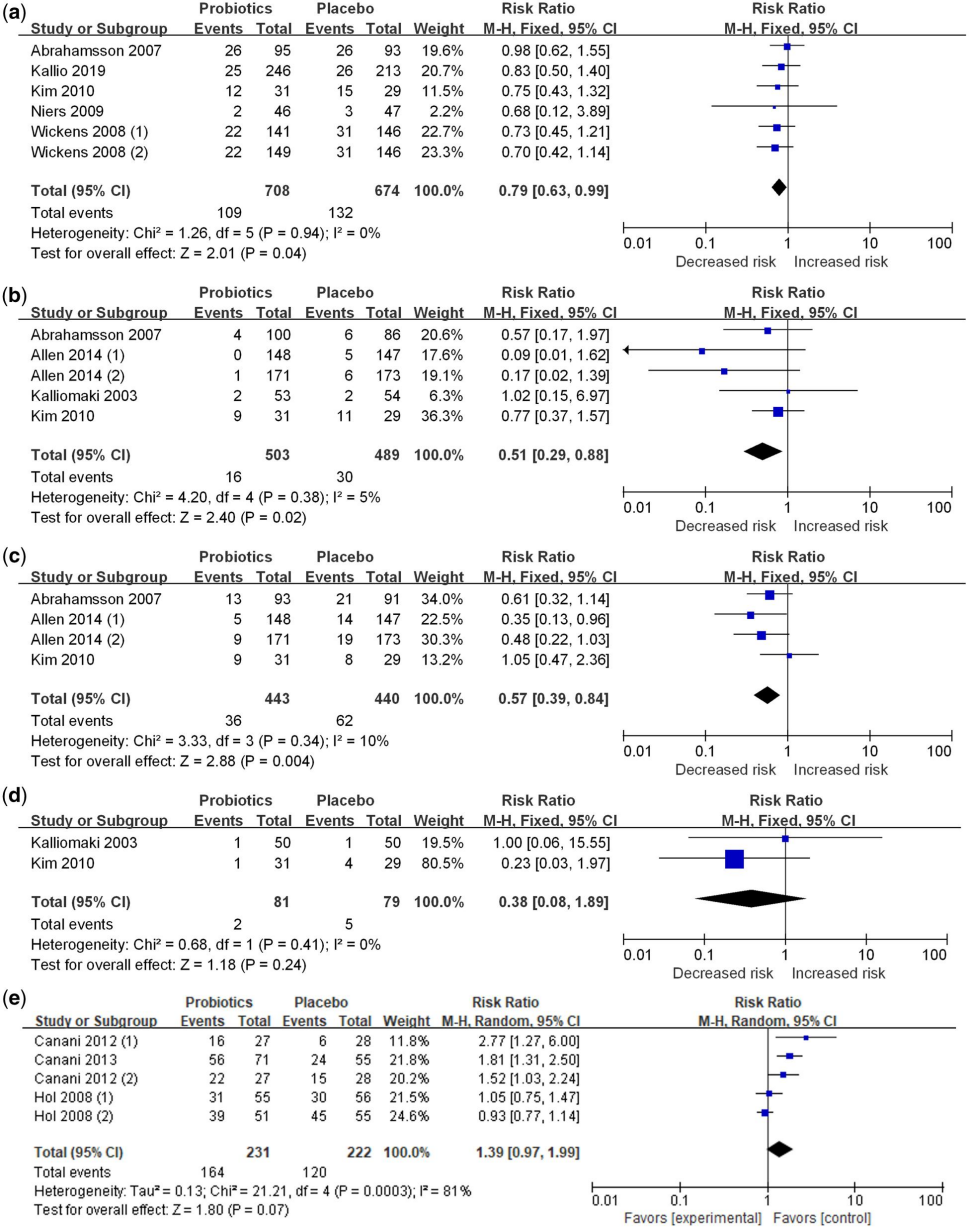
**Forest plots of randomized controlled trials for probiotic supplementation during pregnancy and infancy on (a) total food allergy, (b) cow-milk allergy, (c) egg allergy, and (d) peanut allergy and (e) forest plot of randomized controlled trials for probiotic supplementation on tolerance towards food**. *Abbreviation*: M-H, Mantel-Haenszel.

Seven studies assessed the effects of probiotics supplementation during pregnancy and infancy on food allergies in children.^7^^,^^16–21^ The number of studies associated with food allergies,^16–19^^,^^21^ cow-milk allergy,^7^^,^^16^^,^^19^^,^^20^ egg allergy,^7^^,^^16^^,^^19^ and peanut allergy^19^^,^^20^ were 5, 4, 3, and 2, respectively. Probiotics were provided in capsule or powder forms and at a dose of 0.1 to 24.2 billion colony-forming units (CFUs) per day. Probiotics supplementation during pregnancy and infancy can reduce the risk of total food allergy (relative risk [RR], 0.79; 95% CI, 0.63–0.99; *I^2^* = 0%), cow-milk allergy (RR, 0.51; 95% CI, 0.29–0.88; *I^2^* = 5%), and egg allergy (RR, 0.57; 95% CI, 0.39–0.84; *I^2^* = 10%) (Fig. 2). No significant associations with peanut allergies were observed. Subgroup analysis (Table 5) showed that probiotics supplementation significantly decreased the risk of cow-milk and egg allergies in Europe but not in Oceania and Asia. The use of more than 2 types of probiotics had a beneficial effect on cow-milk allergy (RR, 0.45; 95% CI, 0.23–0.86; *I^2^* = 51.8%) and egg allergy (RR, 0.55; 95% CI, 0.34–0.89; *I^2^* = 40.3%). Based on the dose–response data, probiotics supplementation during pregnancy and infancy decreased the risk of food allergy in a nonlinear fashion (*R^2^* = 0.15, *P-*nonlinearity = 0.23), with stronger effects at doses of approximately 3–12 × 10^9^ CFUs per day (see Fig. S1 in the Supporting Information online). The summary RR (95% CI) for a 1.8 × 10^9^–CFU per day increment was 0.96 (0.93–0.99) for food allergy risk.

**Table 5:** Subgroup analysis of probiotics supplementation during pregnancy and infancy on food allergy in children

	Food allergy					Cow-milk allergy					Egg allergy					Peanut allergy				
Subgroup	n	Pooled RRs (95% CI)		*I^2^*	*P*	n	Pooled RRs (95% CI)		*I^2^*	*P*	n	Pooled RRs (95% CI)		*I^2^*	*P*	n	Pooled RRs (95% CI)		*I^2^*	*P*
Probiotics supplementation during pregnancy and infancy																				
Follow-up duration																				
1∼12 mo	1	0.748	(0.425, 1.317)	—		2	0.545	(0.273, 1.088)	57.3%	0.126	2	0.613	(0.331, 1.136)	65.3%	0.089	1	0.234	(0.028, 1.972)	—	
13∼24 mo	4	0.790	(0.600, 1.041)	0.0%	0.755	2	0.379	(0.134, 1.069)	0.2%	0.317	2	0.546	(0.336, 0.888)	0.0%	0.642	—	—	—	—	
25∼36 mo	—	—	—	—		—	—	—	—		—	—	—	—		—	—	—	—	
>36 mo	1	0.833	(0.496, 1.397)	—		1	1.019	(0.149, 6.970)	—		—	—	—	—		1	1.000	(0.064, 15.548)	—	
Country																				
Europe	3	0.892	(0.635, 1.253)	0.0%	0.854	4	0.363	(0.157, 0.837)	1.3%	0.386	3	0.497	(0.321, 0.768)	0.0%	0.660	1	1.000	(0.064, 15.548)	—	
Oceania	2	0.715	(0.504, 1.015)	0.0%	0.877	—	—	—	—		—	—	—	—		—	—	—	—	
Asia	1	0.748	(0.425, 1.317)	—		1	0.765	(0.372, 1.574)	—		1	1.158	(0.708, 1.896)	—		1	0.234	(0.028, 1.972)	—	
Probiotics type																				
1	3	0.794	(0.600, 1.049)	0.0%	0.561	2	0.678	(0.242, 1.896)	0.0%	0.622	1	0.606	(0.323, 1.136)	—		1	1.000	(0.064, 15.548)	—	
>2	3	0.795	(0.542, 1.164)	0.0%	0.949	3	0.447	(0.231, 0.862)	51.8%	0.126	3	0.552	(0.341, 0.892)	40.3%	0.187	1	0.234	(0.028, 1.972)	—	
Probiotics supplementation during infancy																				
Follow-up duration																				
1∼12 mo	5	1.246	(0.941, 1.652)	0.0%	0.790	4	0.746	(0.478, 1.163)	8.5%	0.351	3	1.291	(0.829, 2.011)	0.0%	0.726	1	1.412	(0.637, 3.130)	—	
13∼24 mo	2	1.192	(0.771, 1.841)	0.0%	0.342	1	0.726	(0.411, 1.284)	—		—	—	—	—		—	—	—	—	
25∼36 mo	2	0.960	(0.791, 1.164)	0.0%	0.615	1	0.194	(0.023, 1.621)	—		1	1.336	(0.572, 3.121)	—		1	1.601	(0.625, 4.103)	—	
>36 mo	2	1.294	(0.712, 2.352)	0.0%	0.383	1	0.592	(0.140, 2.512)	—		1	1.579	(0.418, 5.968)	—		1	2.171	(0.741, 6.365)	—	
Country																				
Europe	6	0.991	(0.843, 1.164)	0.0%	0.969	4	0.681	(0.475, 0.976)	0.0%	0.815	—	—	—	—		—	—	—	—	
Oceania	4	1.500	(1.057, 2.128)	0.0%	0.782	3	0.719	(0.290, 1.783)	48.3%	0.145	3	1.443	(0.948, 2.197)	0.0%	0.974	3	1.642	(0.969, 2.784)	0.0%	0.819
Asia	1	1.138	(0.394, 3.290)	—		—	—	—	—		2	0.976	(0.414, 2.301)	0.0%	1.000	—	—	—	—	
Probiotics type																				
1	4	1.366	(0.985, 1.895)	0.0%	0.708	3	0.719	(0.290, 1.783)	48.3%	0.145	3	1.443	(0.948, 2.197)	0.0%	0.974	3	1.642	(0.969, 2.784)	0.0%	0.819
>2	7	1.009	(0.858, 1.187)	0.0%	0.907	4	0.681	(0.475, 0.976)	0.0%	0.815	2	0.976	(0.414, 2.301)	0.0%	1.000	—	—	—	—	—

*P* values for heterogeneity.

*Abbreviation:* RR, relative risk.

Only 1 study has assessed the effect of probiotics supplementation during pregnancy on food allergies in children.^22^ Probiotics were administered in capsule form at a dose of 1.8 × 10^9^ CFUs per day. There was no significant effect of probiotics supplementation on total food, cow-milk, egg, or peanut allergies.

**Figure 3:**
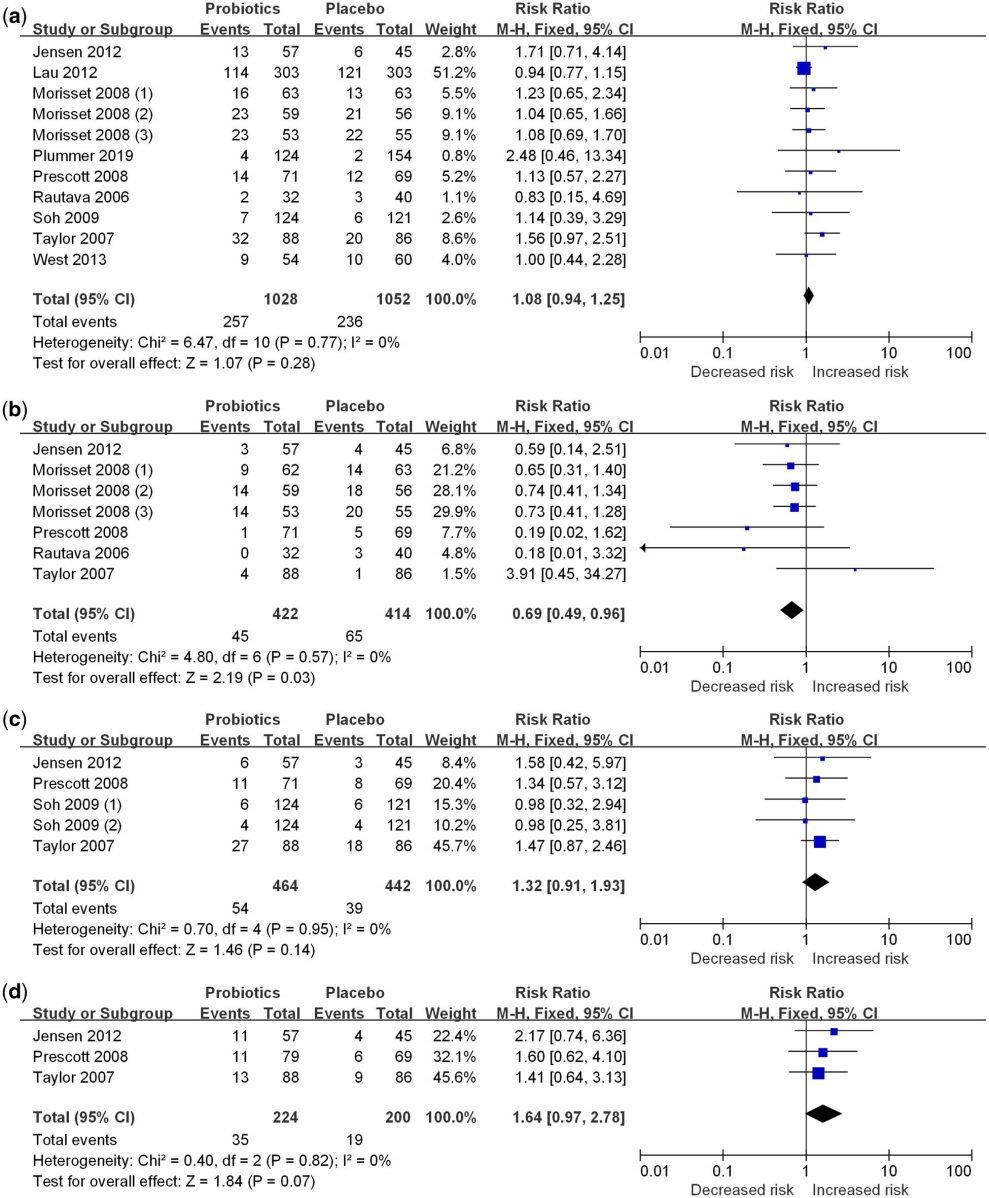
**Forest plots of randomized controlled trials for probiotic supplementation during infancy on (a) total food allergy, (b) cow-milk allergy, (c) egg allergy, and (d) peanut allergy**. *Abbreviation*: M-H, Mantel-Haenszel.

The effects of probiotics supplementation on infants were evaluated in 9 intervention trials,^8^^,^^23–30^ which included 9 for total food allergy,^8^^,^^23–30^ 5 for cow-milk allergy,^8^^,^^24–26^^,^^29^ 4 for egg allergy,^8^^,^^25^^,^^26^^,^^28^ and 3 for peanut allergy.^8^^,^^25^^,^^26^ Probiotics were provided in powder form or as part of formula milk at a dose of 9–305 × 10^7^ CFUs per day. Supplementation of probiotics in infants significantly decreased the risk of cow-milk allergy (RR, 0.69; 95% CI, 0.49–0.96; *I^2^* = 0%) (Fig. 3). There were no significant effects on total food, egg, or peanut allergies. Subgroup analysis (Table 5) showed a significant difference among regions and probiotics types in cow-milk allergies. A significant reduction in risk was observed in the European subgroup (RR, 0.68; 95% CI, 0.48–0.98; *I^2^* = 0%) and in those taking more than 2 types of probiotics (RR, 0.68; 95% CI, 0.48–0.98; *I^2^* = 0%). No association was found in the subgroups of other regions or in 1 type of probiotic. Probiotics dosage (*R^2^* = 0.183, *P-*nonlinearity = 0.1084) and follow-up duration (*R^2^* = 0.183, *P-*nonlinearity = 0.1084) did not show any nonlinear relationship with the risk of food allergy (see Fig. S1 in the Supporting Information online).

Three studies assessed the effects of probiotics supplementation during infancy on tolerance towards cow milk.^31–33^ The results indicated that probiotics supplementation did not accelerate cow-milk tolerance in infants with cow-milk allergy (RR, 1.39; 95% CI, 0.97–1.99; *I^2^* = 81%) (Fig. 2).

### Perturbations of gut microbiota in food-allergic children with age progression

#### Alpha diversity

Data from 11 studies were included in this meta-analysis (308 patients and 655 controls).^34^^,^^36^^,^^38^^,^^40^^,^^41^^,^^44–49^ Four indices were used to assess alpha diversity, including estimates of diversity, richness, and evenness (Shannon, Simpson, Chao1, and observed operational taxonomic units [OTUs]).

Eleven studies reported the Shannon index in patients (n = 308) vs controls (n = 655).^34^^,^^36^^,^^38^^,^^40^^,^^41^^,^^44–49^ No difference was observed between the groups (standardized mean difference [SMD],  −0.13; 95% CI, −0.42 to 0.17; *I^2^* = 81.1%) (see Fig. S2 in the Supporting Information online). Within the follow-up duration categories, there was a significant decrease, with a small effect size in the 0–6-month group (SMD,  −0.36; 95% CI, −0.70, −0.03; *I^2^* = 55%) and an increase in the 6–12-month group (SMD, 0.5; 95% CI, 0.21 to 0.80; *I^2^* = 0%). Three studies provided data on the Simpson index in patients (n = 73) and controls (n = 77).^36^^,^^46^^,^^48^ The pooled estimate did not show any difference between the groups (SMD,  −0.18; 95% CI, −0.74 to 0.38; *I^2^* = 60.8%), except for the 12–48-month subgroup, which showed a decrease (SMD,  −0.74; 95% CI, −1.38, −0.09) (see Fig. S2 in the Supporting Information online).

With regard to richness, 4 studies provided data on Chao1 in patients (n = 125) vs controls (n = 458).^38^^,^^41^^,^^44^^,^^49^ There was no significant difference between the groups (SMD, 0.44; 95% CI, −0.48 to 1.36; *I^2^* = 41.8%) (see Fig. S2 in the Supporting Information online). With regard to evenness, observed OTU data were provided in 4 studies (n = 89 patients, n = 97 controls).^40^^,^^41^^,^^47^^,^^48^ No difference was observed between the groups (SMD,  −0.24; 95% CI, −0.93 to 0.45; *I^2^* = 0%) (see Fig. S2 in the Supporting Information online).

#### Beta diversity

Beta-diversity comparisons between patients with food allergies and healthy controls were reported in 13 studies. Seven studies reported significant differences, whereas 5 studies reported no differences. The remaining study demonstrated a significant difference in infants aged 3–6 months and 13–16 months and a nonsignificant difference in infants aged 7–12 months. These findings provide reliable evidence for differences in the gut microbiota composition of food-allergic children compared with that in the controls. However, this difference appears to be dependent on the age of children.

#### Differentially abundant microbial taxa in children with age progression

**Figure 4:**
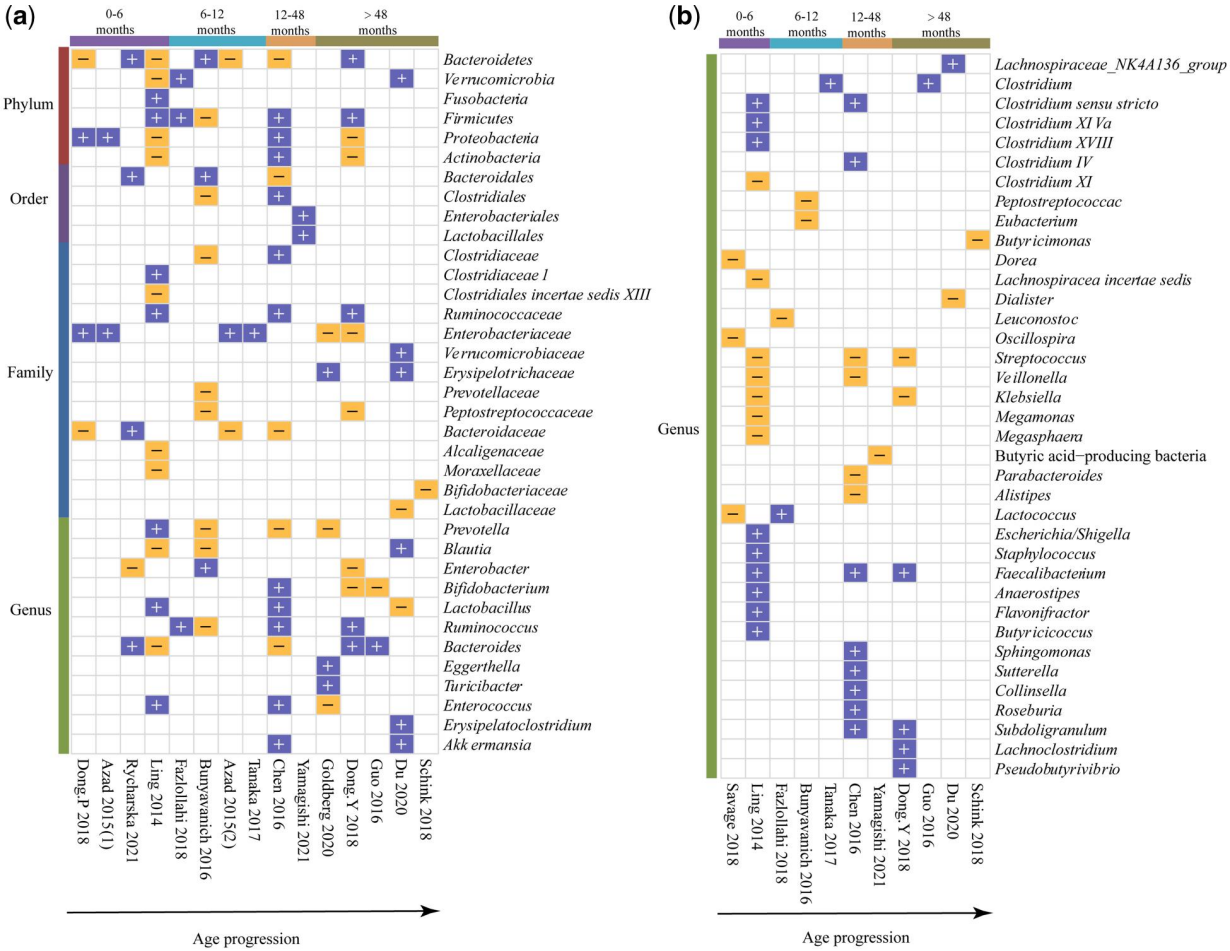
**Differentially abundant microbial taxa in children with age progression**. Blue cells indicate increases and orange cells indicate decreases.

Fifteen studies were included to assess the relative abundance of gut microbiota and to identify significant differences between patients with food allergy and healthy controls at the phylum, order, family, or genus levels. The differences spanned 6 phyla, 4 orders, 14 families, and 49 genera. Figure 4 shows the changes in gut microbiota abundance with age progression in children with food allergies.

At the phylum level, the gut microbiota abundance differed across all age groups. Verrucomicrobia, Fusobacteria, Firmicutes, and Proteobacteria were elevated, and Actinobacteria was decreased in allergic children. Six families, including *Clostridiaceae*, *Ruminococcaceae, Verrucomicrobiaceae*, and *Erysipelotrichaceae*, increased, and 8 families, including *Prevotellaceae, Bacteroidales*, *Peptostreptococcaceae*, *Bifidobacteriaceae*, and *Lactobacillaceae*, decreased in the stools of children with allergies. At the genus level, the results showed an upward trend for *Prevotella* in allergic infants aged 0–6 months and a downward trend in infants more than 6 months old. The abundances of *Blautia* and *Bacteroides* first decreased and then increased with age. The abundance of probiotics in the intestine, including *Lactobacillus*, *Bifidobacterium*, and *Enterococcus*, decreases after age 48 months in children. Twenty-four genera, including *Ruminococcus*, *Clostridium*, *Eggerthella*, *Flavonifractor*, *Anaerostipes*, and *Faecalibacterium*, were elevated. The other 17 genera, including *Butyricicoccus*, *Butyricimonas*, *Leuconostoc*, *Veillonella*, and butyric acid–producing bacteria (eg, *Eubacterium*, *Dialister*), decreased with food allergy.

### Probiotics supplementation on gut microbiota

Two studies on the effect of probiotics supplementation during pregnancy and infancy on gut microbiota composition were included.^17^^,^^18^ Wickens et al^17^ suggested that bacteria from the environment can be inoculated into the digestive tract at birth, such as *Bifidobacterium animalis* subsp*. lacti*s and *Lactobacillus rhamnosus.* However, children in the probiotics group had significantly higher detection rates of probiotics in feces at 3, 12, and 24 months than those in the placebo group.^17^  *L. rhamnosus* was more likely to be present in feces at 3 months (71.5%) than *B. animalis* subsp*. lactis* (22.6%), but the detection rate was similar at 24 months.^17^ Niers et al^18^ showed that, within the first 3 months of life, *Lactococcus lactis* colonization was significantly more frequent and more numerous in the probiotics group than in the placebo group; all children in the probiotics group and 85% of children in the placebo group had bifidobacteria colonization.

Two studies on the effect of probiotics supplementation during infancy on the gut microbiota composition were included.^25^^,^^27^ Taylor et al^25^ suggested that, at 6 months of age, the rate of *Lactobacillus* colonization was significantly higher in the probiotics group (36%) than in the placebo group (21.6%); however, *Lactobacillus* colonization was associated with an increased risk of milk sensitization. West et al^27^ concluded that *Lactobacillus paracasei* ssp*. paracasei* F19 (LF19) is a transient colonizer because LF19 was not detected in fecal samples from children aged 8–9 years.

## DISCUSSION

Proactive approaches are required to reduce the global burden of food allergies. The findings from this current meta-analysis and review suggested that probiotics supplementation during pregnancy and infancy could reduce the risk of total food, cow-milk, and egg allergy, and probiotics supplementation during infancy reduced the risk of milk allergy in children. Dose–response analysis indicated that more than 2 types of probiotics species had beneficial effects, and an increasement of 1.8 × 10^9^ CFUs of probiotics during pregnancy and infancy per day could reduce the risk of food allergy by 4%. The age-related microbiota composition can partly account for the varied effects of probiotics on food allergies. In this study, we explored for the first time the different effects of probiotics during pregnancy or infancy on various food allergies and probed the influencing factors, first revealing the role played by age-dependent microbiota perturbations.

The decreased risk of cow-milk allergy associated with probiotics in the meta-analysis is in concordance with findings from a systematic review of observational studies.^14^ Nevertheless, no meta-analysis has differentiated the effects of probiotics supplementation during pregnancy or infancy on distinct types of food allergies in children. Additionally, while the majority of studies in this meta-analysis indicated no significant impact of probiotics supplementation on food allergies, the overall results of the meta-analysis demonstrated a reduction in the risk of total food allergy, cow-milk allergy, and egg allergy associated with probiotics supplementation during pregnancy and infancy. One potential explanation could be that the meta-analysis expanded the sample size by aggregating multiple studies to make the protective effect of probiotics more apparent.

**Figure 5:**
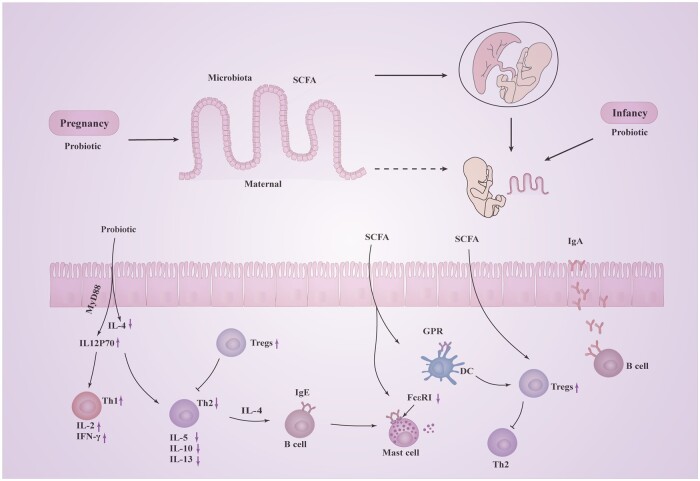
**Schematic mechanisms by which probiotic and gut bacteria modulate food allergy in children**. *Abbreviations*: DC, dendritic cell; FcεRI, Fc-epsilon receptor I; GPR, G protein–coupled receptor; IFN-γ, interferon-γ; IgA, immunoglobulin A; IgE, immunoglobulin E; IL, interleukin; SCFA, short-chain fatty acid; Th, T-helper; Tregs, regulatory T cells.

Probiotics supplementation during both pregnancy and infancy had a more favorable effect on food allergies compared with supplementation during either pregnancy or infancy alone. In this meta-analysis and review, the supplementation of probiotics during both pregnancy and infancy was associated with a decreased risk of total food allergy, cow-milk allergy, and egg allergy. Meanwhile, probiotics supplementation during infancy alone was linked to a reduced risk of milk allergy, while supplementation during pregnancy alone showed no effect on food allergy. Although direct oral supplementation of probiotics during infancy may affect immune development through allergen-specific mechanisms, concurrent probiotics supplementation during pregnancy and infancy may have broader effects on the developing immune system.^14^ First, it may be related to the development of the infant immune system, which is influenced by maternal cells, pathogens, and commensal microorganisms (Fig. 5). Probiotics supplementation in pregnant mothers, which may directly modulate intestinal microorganisms, can affect the number of induced cells in the fetal lymphoid tissue and the development of secondary lymphoid organs.^51^ Probiotics colonize the intestine and ferment dietary fibers into short-chain fatty acids (SCFAs), which, in turn, can foster immune maturation in offspring by activating G protein–coupled receptors (GPRs) on the surface of the immune cells.^51^ Previous studies have shown that probiotics fermentation of dietary fiber during pregnancy and lactation can induce the differentiation of regulatory T cells in the offspring.^52^ Second, maternal gut microbes can influence the composition and function of offspring gut microbes, thus promoting maturation of the body's immune system (Fig. 5). A prospective birth cohort study including 70 mother–infant pairs suggested that maternal gut microbiota share genes with infant gut microbiota from the perinatal period until a few weeks after birth and can shape the infant gut microbiota through horizontal gene transfer.^53^ Newborns are born in symbiosis with microorganisms before birth and have access to oral and body surface microorganisms through birth to promote the growth and maturation of the immune system.^51^ Third, prolonged and higher-dose probiotic supplementation during both pregnancy and infancy is more conducive to the colonization of beneficial bacteria in the intestinal tract.

The present study demonstrated that supplementing with more than 2 types of probiotics had a preventive effect on egg and milk allergies, whereas a single type did not. Combining more than 2 types of probiotics helps colonize the intestine more easily than a single type of probiotic. The synergistic effects of multiple probiotics can also modulate the immune responses.^54^ The combined use of several types of probiotics in fish significantly enhanced beneficial intestinal bacterial counts and mucosal immune responses, and altered the expression of interleukin (IL)-1β, tumor necrosis factor α (TNF-α), and IL-10 cytokines.^54^ The combination of *Bifidobacterium*, *Lactobacillus*, and *Enterococcus* significantly increases the richness and evenness of the gut microbiota in mice,^55^ which may contribute to the reduction in food allergies.^45^ The approach of colonization of bacteria from a healthy population without food allergies is more advantageous than using a single strain. Another study found that the colonization of healthy infants by gut bacteria can protect against cow-milk allergy in germ-free mice.^56^ Therefore, targeting underlying microbiota dysbiosis could be an option for reducing food allergies in the future.

The current discourse on the impact of probiotics supplementation on allergic disease symptoms remains contentious. While probiotics supplementation did not accelerate cow-milk tolerance in infants with cow-milk allergy in this work, another study involving *Lactobacillus rhamnosus* GG (LGG) supplementation in mothers of breastfed infants with cow-milk allergy significantly improved Scoring Atopic Dermatitis (SCORAD) scores.^57^^,^^58^ Previous research suggests that probiotic supplementation can alleviate eczema and atopic dermatitis (AD) symptoms, resulting in reduced SCORAD scores and shorter eczema treatment duration.^59^ In a 12-week clinical trial involving 50 children with moderate AD, the administered probiotic mixture effectively lowered the SCORAD scores and reduced topical steroid use.^60^ However, not all clinical studies support a clear role for probiotics in alleviating allergic disease symptoms. A 6-week probiotic mixture supplementation study in 100 children with mild-to-moderate AD did not reveal additional therapeutic or immunomodulatory effects of probiotics on AD treatment.^61^ Some studies reported side effects linked to specific opportunistic bacterial pathogens that should be avoided.^58^^,^^59^ The differential impact of probiotics is associated with the type or strain, dosage, and equilibrium of the patient's gut microbiota.^59^ Additionally, an RCT with 49 participants revealed variations in the consumption of probiotic *Bifidobacterium lactis* V9 among subjects with differing gut microbiota.^62^ Therefore, individual differences emerge as a pivotal factor influencing outcomes, underscoring the need for larger studies to validate these results in the future.

The balance of gut microbiota is closely related to food allergies, and the intestinal microbiota composition differs across age groups in infants and children. As infants grow and develop, the gut microbial composition in individuals tends to be distributed toward an adult-like microbiota with a higher richness.^63^^,^^64^ In the early months of life, infants are exposed to a variety of microbial species, some of which colonize the intestines.^65^ Breast milk can provide infants with more probiotics, such as bifidobacteria and lactobacilli, and prebiotics, such as oligosaccharides, making it easier to form a bifidobacteria-dominated microbiota.^66^^,^^67^ Upon the introduction of solid foods, the intestinal microbiota undergoes a transition from infancy to adulthood, gradually adopting a microbiota dominated by *Bacteroides* and *Firmicutes* genera.^68^ The abundances of intestinal *Bacteroides* in food-allergic infants were inconsistent compared with healthy controls, suggesting that the intestinal flora composition of food-allergic children differed from that of normal children but tended to resemble that of adults. The abundance of 3 probiotics—*Lactobacillus*, *Bifidobacterium*, and *Enterococcus*—decreased after 48 months of age in children with food allergies compared with healthy controls. The elevated abundance of probiotics plays an essential role in regulating gut microbiota. Probiotics colonize the intestine and interact with intestinal epithelial cells or produce active substances to alleviate food-allergic diseases.^69^^,^^70^  *Prevotella copri* is a major species of the *Prevotella* genus in the human intestinal microbiome.^71^ It is responsible for the production of succinate, which stimulates dendritic cell (DC) function and migration.^72^ The establishment of a fetal DC network provides protection against allergic diseases. The decreased abundance of *Veillonella* in saliva and stool samples is significantly related to peanut allergy.^73^^,^^74^  *Veillonella* can reduce inflammatory responses by degrading amylase-trypsin inhibitor variants and alleviating the symptoms of wheat food allergy.^75^

Continuous probiotics supplementation in infants during the critical period of microbiota and immune system maturation may offer benefits. Two mechanisms of action for probiotics supplementation have been proposed (Fig. 5). First, probiotics supplementation may improve food-allergic diseases by rebalancing the T-helper (Th) 1/Th2 immune response.^76^ Enhanced Th2 immune responses are associated with food allergies,^77^ with IL-4 directing Th2 cell development. Additionally, Th2 cell differentiation induces the production of various cytokines, including IL-5, IL-10, and IL-13.^78^ IL-12p70 is a major cell-mediated Th1 immune cytokine promoting the production of interferon-γ (IFN-γ) and IL-2.^79^^,^^80^ Intervention with *Bifidobacterium breve* M-16V may enhance Th1-related cytokine IL-12p70 production by activating MyD88 expression and decreasing Th2 cell proportion.^81^  *Lactobacillus reuteri* Fn041 regulates adaptive immune responses, leading to increases in the levels of IFN-γ and IL-12 associated with Th1, as well as reducing the IL-4 and IgE levels associated with Th2 to suppress the inflammatory infiltration of eosinophils and mast cells.^82^  *L. reuteri* Fn041 can improve early atopic immune responses by regulating the balance between Th1 and Th2 immune responses.^82^

Second, the intestinal microbiota, engaged in complex metabolic activities, generates numerous metabolites in the body (Fig. 5). The abundances of *Prevotella* and *Veillonella* were significantly correlated with the production of SCFAs in the oral and intestinal microbiota.^74^ Butyric acid–producing bacteria, including *Faecalibacterium*, *Anaerostipes*, and *Eubacterium* (effective SCFA producers),^83–85^ were decreased in this study. SCFAs binding to GPRs can activate diverse signaling cascades.^86^ SCFA receptors are expressed on the surface of DCs and regulatory T cells (Tregs). SCFAs enhance the function of intestinal CD103^+^ DCs by stimulating GPR109a cell surface receptor.^87^ The DC subpopulation can trigger the proliferation and expansion of Tregs in mesenteric lymph nodes, thereby promoting tolerance in the intestine.^87^ SCFAs increased the number of IgA-secreting lamina propria plasma cells and B cells in Peyer's patches to protect against food allergy.^88^^,^^89^ SCFAs also directly inhibit mast cell degranulation and histamine release, regulating FcεRI-mediated signaling molecules to alleviate allergic responses.^90^

A strength of our meta-analysis is the novel exploration of the distinct effects of probiotics during pregnancy or infancy on various food allergies, probing the influencing factors and, foremost, revealing the role played by age-dependent microbiota perturbations.

However, our study had several limitations. There is a paucity of experimental evidence regarding the effect of probiotics supplementation on food allergies, especially peanut allergies. Differences in probiotics and their mixtures used in these studies could introduce bias into the results.

## CONCLUSION

Probiotics supplementation during both pregnancy and infancy demonstrated greater effectiveness against food allergies compared with supplementation during either pregnancy or infancy alone, with enhanced benefits observed at higher doses. Children with food allergies exhibited age-related changes in microbial profiles, offering insight into the varying effects of probiotics on food allergies. This study introduces a novel perspective to the prevention and treatment of food allergies in children.

Despite numerous conducted studies, additional trials with larger sample sizes are required to assess the effects of diverse probiotic strains, dosages, and intervention durations on food allergies. This systematic review and meta-analysis suggested that probiotics supplementation may offer significant benefits to children with food allergies. The potential mechanism might involve modifying the composition of gut microbiota. In future research, it is crucial to further explore the factors influencing gut microbiota stabilization. Additionally, conducting gut microbiota testing before probiotic intervention could enhance its effectiveness.

## Supplementary Material

nuae024_Supplementary_Data

## Supporting Information

The following Supporting Information is available through the online version of this article at the publisher’s website.

***Material S1* Search strategy**

***Figure S1* Nonlinear dose-responses between (a) probiotic supplementation during pregnancy and infancy, (b) probiotic supplementation during infancy and the risk ratio in food allergy, and (c) nonlinear dose-response between follow-up duration of probiotic supplementation during infancy and the risk ratio in food allergy. Shaded regions represented the 95% CIs**

***Figure S2* Forest plots of case-control studies for gut microbiota of alpha diversity in children with food allergy. Diversity including (a) Shannon index and (b) Simpson index, and richness including (c) Chao 1 and (d) evenness including observed OTUs**

***Table S1* The measures of collection, preservation, and extraction of intestinal samples and the methods of beta-diversity analysis**

***Table S2* Quality assessment of studies investigating probiotics supplementation during pregnancy and infancy on food allergy**

***Table S3* Quality assessment of studies investigating perturbations of gut microbiota composition with food allergy in children**

***Table S4* PRISMA 2020 checklist**

## References

[1] Spolidoro GCI , Tesfaye Amera Y, Ali MM, et al Frequency of food allergy in Europe: an updated systematic review and meta-analysis. Allergy. 2022;78:351–368. 36271775 10.1111/all.15560 PMC10099188

[2] Warren C , Bartell T, Nimmagadda SR, et al Socioeconomic determinants of food allergy burden: a clinical introduction. Ann Allergy Asthma Immunol. 2022;129:407–416. 35914663 10.1016/j.anai.2022.07.021

[3] Sindher SB , Long A, Chin AR, et al Food allergy, mechanisms, diagnosis and treatment: innovation through a multi-targeted approach. Allergy. 2022;77:2937–2948. 35730331 10.1111/all.15418

[4] Joseph CL , Zoratti EM, Ownby DR, et al Exploring racial differences in IgE-mediated food allergy in the WHEALS birth cohort. Ann Allergy Asthma Immunol. 2016;116:219–224.e1. 26837607 10.1016/j.anai.2015.12.019 PMC4864956

[5] Caminero A , Meisel M, Jabri B, et al Mechanisms by which gut microorganisms influence food sensitivities. Nat Rev Gastroenterol Hepatol. 2019;16:7–18. 30214038 10.1038/s41575-018-0064-z PMC6767923

[6] Deak PE , Vrabel MR, Kiziltepe T, et al Determination of crucial immunogenic epitopes in major peanut allergy protein, Ara h2, via novel nanoallergen platform. Sci Rep. 2017;7:3981. 28638052 10.1038/s41598-017-04268-6 PMC5479826

[7] Allen SJ , Jordan S, Storey M, et al Probiotics in the prevention of eczema: a randomised controlled trial. Arch Dis Child. 2014;99:1014–1019. 24947281 10.1136/archdischild-2013-305799 PMC4215350

[8] Jensen MP , Meldrum S, Taylor AL, et al Early probiotic supplementation for allergy prevention: long-term outcomes. J Allergy Clin Immunol. 2012;130:1209–1211.e5. 22958946 10.1016/j.jaci.2012.07.018

[9] Olin A , Henckel E, Chen Y, et al Stereotypic immune system development in newborn children. Cell. 2018;174:1277–1292.e14. 30142345 10.1016/j.cell.2018.06.045 PMC6108833

[10] Park JE , Jardine L, Gottgens B, et al Prenatal development of human immunity. Science. 2020;368:600–603. 32381715 10.1126/science.aaz9330 PMC7612900

[11] Beller L , Deboutte W, Falony G, et al Successional stages in infant gut microbiota maturation. mBio. 2021;12:e0185721. 34903050 10.1128/mBio.01857-21 PMC8686833

[12] Chun Y , Grishin A, Rose R, et al Longitudinal dynamics of the gut microbiome and metabolome in peanut allergy development. J Allergy Clin Immunol. 2023;152:1569–1580. 37619819 10.1016/j.jaci.2023.08.012 PMC11440358

[13] Colquitt AS , Miles EA, Calder PC. Do probiotics in pregnancy reduce allergies and asthma in infancy and childhood? A systematic review. Nutrients. 2022;14:1852. 35565819 10.3390/nu14091852 PMC9105059

[14] Garcia-Larsen V , Ierodiakonou D, Jarrold K, et al Diet during pregnancy and infancy and risk of allergic or autoimmune disease: a systematic review and meta-analysis. PLoS Med. 2018;15:e1002507. 29489823 10.1371/journal.pmed.1002507 PMC5830033

[15] Cait A , Wedel A, Arntz JL, et al Prenatal antibiotic exposure, asthma, and the atopic march: a systematic review and meta-analysis. Allergy. 2022;77:3233–3248. 35689800 10.1111/all.15404

[16] Abrahamsson TR , Jakobsson T, Böttcher MF, et al Probiotics in prevention of IgE-associated eczema: a double-blind, randomized, placebo-controlled trial. J Allergy Clin Immunol. 2007;119:1174–1180. 17349686 10.1016/j.jaci.2007.01.007

[17] Wickens K , Black PN, Stanley TV, et al; Probiotic Study Group. A differential effect of 2 probiotics in the prevention of eczema and atopy: a double-blind, randomized, placebo-controlled trial. J Allergy Clin Immunol. 2008;122:788–794. 18762327 10.1016/j.jaci.2008.07.011

[18] Niers L , Martín R, Rijkers G, et al The effects of selected probiotic strains on the development of eczema (the PandA study). Allergy. 2009;64:1349–1358. 19392993 10.1111/j.1398-9995.2009.02021.x

[19] Kim JY , Kwon JH, Ahn SH, et al Effect of probiotic mix (Bifidobacterium bifidum, Bifidobacterium lactis, Lactobacillus acidophilus) in the primary prevention of eczema: a double-blind, randomized, placebo-controlled trial. Pediatr Allergy Immunol. 2010;21:386–393. 10.1111/j.1399-3038.2009.00958.x 19840300

[20] Kalliomäki M , Salminen S, Poussa T, et al Probiotics and prevention of atopic disease: 4-year follow-up of a randomised placebo-controlled trial. Lancet. 2003;361:1869–1871. 12788576 10.1016/S0140-6736(03)13490-3

[21] Kallio S , Kukkonen AK, Savilahti E, et al Perinatal probiotic intervention prevented allergic disease in a caesarean-delivered subgroup at 13-year follow-up. Clin Exp Allergy. 2019;49:506–515. 30472801 10.1111/cea.13321

[22] Boyle RJ , Ismail IH, Kivivuori S, et al Lactobacillus GG treatment during pregnancy for the prevention of eczema: a randomized controlled trial. Allergy. 2011;66:509–516. 21121927 10.1111/j.1398-9995.2010.02507.x

[23] Plummer EL , Chebar Lozinsky A, Tobin JM, et al; ProPrems Study Group. Postnatal probiotics and allergic disease in very preterm infants: sub-study to the ProPrems randomized trial. Allergy. 2020;75:127–136. 31608448 10.1111/all.14088

[24] Morisset M , Aubert-Jacquin C, Soulaines P, et al A non-hydrolyzed, fermented milk formula reduces digestive and respiratory events in infants at high risk of allergy. Eur J Clin Nutr. 2011;65:175–183. 21081959 10.1038/ejcn.2010.250

[25] Taylor AL , Dunstan JA, Prescott SL. Probiotic supplementation for the first 6 months of life fails to reduce the risk of atopic dermatitis and increases the risk of allergen sensitization in high-risk children: a randomized controlled trial. J Allergy Clin Immunol. 2007;119:184–191. 17208600 10.1016/j.jaci.2006.08.036

[26] Prescott SL , Wiltschut J, Taylor A, et al Early markers of allergic disease in a primary prevention study using probiotics: 2.5-year follow-up phase. Allergy. 2008;63:1481–1490. 18925885 10.1111/j.1398-9995.2008.01778.x

[27] West CE , Hammarström ML, Hernell O. Probiotics in primary prevention of allergic disease—follow-up at 8-9 years of age. Allergy. 2013;68:1015–1020. 23895631 10.1111/all.12191

[28] Soh SE , Aw M, Gerez I, et al Probiotic supplementation in the first 6 months of life in at risk Asian infants—effects on eczema and atopic sensitization at the age of 1 year. Clin Exp Allergy. 2009;39:571–578. 19134020 10.1111/j.1365-2222.2008.03133.x

[29] Rautava S , Arvilommi H, Isolauri E. Specific probiotics in enhancing maturation of IgA responses in formula-fed infants. Pediatr Res. 2006;60:221–224. 16864708 10.1203/01.pdr.0000228317.72933.db

[30] Lau S , Gerhold K, Zimmermann K, et al Oral application of bacterial lysate in infancy decreases the risk of atopic dermatitis in children with 1 atopic parent in a randomized, placebo-controlled trial. J Allergy Clin Immunol. 2012;129:1040–1047. 22464674 10.1016/j.jaci.2012.02.005

[31] Hol J , van Leer EH, Elink Schuurman BE, et al; Cow's Milk Allergy Modified by Elimination and Lactobacilli Study Group. The acquisition of tolerance toward cow's milk through probiotic supplementation: a randomized, controlled trial. J Allergy Clin Immunol. 2008;121:1448–1454. 18436293 10.1016/j.jaci.2008.03.018

[32] Berni Canani R , Nocerino R, Terrin G, et al Effect of Lactobacillus GG on tolerance acquisition in infants with cow's milk allergy: a randomized trial. J Allergy Clin Immunol. 2012;129:580–582. 22078573 10.1016/j.jaci.2011.10.004

[33] Berni Canani R , Nocerino R, Terrin G, et al Formula selection for management of children with cow's milk allergy influences the rate of acquisition of tolerance: a prospective multicenter study. J Pediatr. 2013;163:771–777.e1. 23582142 10.1016/j.jpeds.2013.03.008

[34] Bao R , Hesser LA, He Z, et al Fecal microbiome and metabolome differ in healthy and food-allergic twins. J Clin Invest. 2021;131:e141935. 33463536 10.1172/JCI141935 PMC7810484

[35] Bunyavanich S , Shen N, Grishin A, et al Early-life gut microbiome composition and milk allergy resolution. J Allergy Clin Immunol. 2016;138:1122–1130. 27292825 10.1016/j.jaci.2016.03.041 PMC5056801

[36] Schink M , Konturek PC, Tietz E, et al Microbial patterns in patients with histamine intolerance. J Physiol Pharmacol. 2018;69:69. 10.26402/jpp.2018.4.09 30552302

[37] Du Z , Gao X, Yin J. Gut microbiome alterations in patients with wheat-dependent exercise-induced anaphylaxis. Int Immunopharmacol. 2020;84:106557. 32388491 10.1016/j.intimp.2020.106557

[38] Fazlollahi M , Chun Y, Grishin A, et al Early-life gut microbiome and egg allergy. Allergy. 2018;73:1515–1524. 29318631 10.1111/all.13389 PMC6436531

[39] Goldberg MR , Mor H, Magid Neriya D, et al Microbial signature in IgE-mediated food allergies. Genome Med. 2020;12:92. 33109272 10.1186/s13073-020-00789-4 PMC7592384

[40] Kourosh A , Luna RA, Balderas M, et al Fecal microbiome signatures are different in food-allergic children compared to siblings and healthy children. Pediatr Allergy Immunol. 2018;29:545–554. 29624747 10.1111/pai.12904

[41] Lee KH , Guo J, Song Y, et al Dysfunctional gut microbiome networks in childhood IgE-mediated food allergy. Int J Mol Sci. 2021;22:2079. 33669849 10.3390/ijms22042079 PMC7923212

[42] Dong Y , Fei P, Han Y, et al Characterization of fecal microbiota, short-chain fatty acids and lactic acid concentrations in 5-8-year-old children with cow milk protein allergy. Iran J Pediatr. 2018;28:e64638.

[43] Guo L , Bai H, Dong Y, et al Comparative analysis of fecal microbiota in 5–8-year-old children with and without cow milk protein allergy. Iran J Pediatr. 2016;26:e6397.

[44] Savage JH , Lee-Sarwar KA, Sordillo J, et al A prospective microbiome-wide association study of food sensitization and food allergy in early childhood. Allergy. 2018;73:145–152. 28632934 10.1111/all.13232 PMC5921051

[45] Dong P , Feng JJ, Yan DY, et al Early-life gut microbiome and cow's milk allergy—a prospective case–control 6-month follow-up study. Saudi J Biol Sci. 2018;25:875–880. 30108435 10.1016/j.sjbs.2017.11.051 PMC6088111

[46] Ling Z , Li Z, Liu X, et al Altered fecal microbiota composition associated with food allergy in infants. Appl Environ Microbiol. 2014;80:2546–2554. 24532064 10.1128/AEM.00003-14 PMC3993190

[47] Łoś-Rycharska E , Gołębiewski M, Sikora M, et al A combined analysis of gut and skin microbiota in infants with food allergy and atopic dermatitis: a pilot study. Nutrients. 2021;13:1682. 34063398 10.3390/nu13051682 PMC8156695

[48] Yamagishi M , Akagawa S, Akagawa Y, et al Decreased butyric acid-producing bacteria in gut microbiota of children with egg allergy. Allergy. 2021;76:2279–2282. 33650199 10.1111/all.14795

[49] Azad MB , Konya T, Guttman DS, et al; CHILD Study Investigators. Infant gut microbiota and food sensitization: associations in the first year of life. Clin Exp Allergy. 2015;45:632–643. 25599982 10.1111/cea.12487

[50] Tanaka M , Korenori Y, Washio M, et al Signatures in the gut microbiota of Japanese infants who developed food allergies in early childhood. FEMS Microbiol Ecol. 2017;93:93. 10.1093/femsec/fix099 28903469

[51] Ganal-Vonarburg SC , Hornef MW, Macpherson AJ. Microbial-host molecular exchange and its functional consequences in early mammalian life. Science. 2020;368:604–607. 32381716 10.1126/science.aba0478

[52] Nakajima A , Kaga N, Nakanishi Y, et al Maternal high fiber diet during pregnancy and lactation influences regulatory T cell differentiation in offspring in mice. J Immunol. 2017;199:3516–3524. 29021375 10.4049/jimmunol.1700248

[53] Vatanen T , Jabbar KS, Ruohtula T, et al Mobile genetic elements from the maternal microbiome shape infant gut microbial assembly and metabolism. Cell. 2022;185:4921–4936.e15. 36563663 10.1016/j.cell.2022.11.023 PMC9869402

[54] Giri SS , Kim HJ, Kim SG, et al Effects of dietary Lactiplantibacillus plantarum subsp. plantarum L7, alone or in combination with Limosilactobacillus reuteri P16, on growth, mucosal immune responses, and disease resistance of Cyprinus carpio. Probiotics Antimicrob Proteins. 2021;13:1747–1758. 34365579 10.1007/s12602-021-09820-5

[55] Huang C , Sun Y, Liao SR, et al Suppression of berberine and probiotics (in vitro and in vivo) on the growth of colon cancer with modulation of gut microbiota and butyrate production. Front Microbiol. 2022;13:869931. 35572672 10.3389/fmicb.2022.869931 PMC9096942

[56] Feehley T , Plunkett CH, Bao R, et al Healthy infants harbor intestinal bacteria that protect against food allergy. Nat Med. 2019;25:448–453. 30643289 10.1038/s41591-018-0324-z PMC6408964

[57] Majamaa H , Isolauri E. Probiotics: a novel approach in the management of food allergy. J Allergy Clin Immunol. 1997;99:179–185. 9042042 10.1016/s0091-6749(97)70093-9

[58] del Giudice MM , Leonardi S, Maiello N, et al Food allergy and probiotics in childhood. J Clin Gastroenterol. 2010;44(Suppl 1):S22–25. 20562632 10.1097/MCG.0b013e3181e102a7

[59] Lopez-Santamarina A , Gonzalez EG, Lamas A, et al Probiotics as a possible strategy for the prevention and treatment of allergies. A narrative review. Foods. 2021;10:701. 33806092 10.3390/foods10040701 PMC8064452

[60] Navarro-Lopez V , Ramirez-Bosca A, Ramon-Vidal D, et al Effect of oral administration of a mixture of probiotic strains on SCORAD index and use of topical steroids in young patients with moderate atopic dermatitis: a randomized clinical trial. JAMA Dermatol. 2018;154:37–43. 29117309 10.1001/jamadermatol.2017.3647 PMC5833582

[61] Yang HJ , Min TK, Lee HW, et al Efficacy of probiotic therapy on atopic dermatitis in children: a randomized, double-blind, placebo-controlled trial. Allergy Asthma Immunol Res. 2014;6:208–215. 24843795 10.4168/aair.2014.6.3.208 PMC4021238

[62] Ma C , Huo D, You Z, et al Differential pattern of indigenous microbiome responses to probiotic Bifidobacterium lactis V9 consumption across subjects. Food Res Int. 2020;136:109496. 32846577 10.1016/j.foodres.2020.109496

[63] Avershina E , Storro O, Oien T, et al Major faecal microbiota shifts in composition and diversity with age in a geographically restricted cohort of mothers and their children. FEMS Microbiol Ecol. 2014;87:280–290. 24112053 10.1111/1574-6941.12223

[64] Korpela K , de Vos WM. Early life colonization of the human gut: microbes matter everywhere. Curr Opin Microbiol. 2018;44:70–78. 30086431 10.1016/j.mib.2018.06.003

[65] Rey-Marino A , Francino MP. Nutrition, gut microbiota, and allergy development in infants. Nutrients. 2022;14:4316. 36297000 10.3390/nu14204316 PMC9609088

[66] Yang B , Chen Y, Stanton C, et al Bifidobacterium and Lactobacillus composition at species level and gut microbiota diversity in infants before 6 weeks. Int J Mol Sci. 2019;20:3306. 31284413 10.3390/ijms20133306 PMC6650860

[67] Favier CF , Vaughan EE, De Vos WM, et al Molecular monitoring of succession of bacterial communities in human neonates. Appl Environ Microbiol. 2002;68:219–226. 11772630 10.1128/AEM.68.1.219-226.2002 PMC126580

[68] Valles Y , Artacho A, Pascual-Garcia A, et al Microbial succession in the gut: directional trends of taxonomic and functional change in a birth cohort of Spanish infants. PLoS Genet. 2014;10:e1004406. 24901968 10.1371/journal.pgen.1004406 PMC4046925

[69] Etyemez Buyukdeveci M , Cengizler I, Balcazar JL, et al Effects of two host-associated probiotics Bacillus mojavensis B191 and Bacillus subtilis MRS11 on growth performance, intestinal morphology, expression of immune-related genes and disease resistance of Nile tilapia (Oreochromis niloticus) against Streptococcusiniae. Dev Comp Immunol. 2023;138:104553. 36122732 10.1016/j.dci.2022.104553

[70] Yan B , Han J, Sun Y, et al Probiotics ameliorate growth retardation of glyphosate by regulating intestinal microbiota and metabolites in crucian carp (Carassius auratus). Sci Total Environ. 2022;851:158260. 36030870 10.1016/j.scitotenv.2022.158260

[71] Gao Y , Nanan R, Macia L, et al The maternal gut microbiome during pregnancy and offspring allergy and asthma. J Allergy Clin Immunol. 2021;148:669–678. 34310928 10.1016/j.jaci.2021.07.011

[72] Rubic T , Lametschwandtner G, Jost S, et al Triggering the succinate receptor GPR91 on dendritic cells enhances immunity. Nat Immunol. 2008;9:1261–1269. 18820681 10.1038/ni.1657

[73] Zhang L , Chun Y, Ho HE, et al Multiscale study of the oral and gut environments in children with high- and low-threshold peanut allergy. J Allergy Clin Immunol. 2022;150:714–720.e2. 35550149 10.1016/j.jaci.2022.04.026 PMC9463091

[74] Ho HE , Chun Y, Jeong S, et al Multidimensional study of the oral microbiome, metabolite, and immunologic environment in peanut allergy. J Allergy Clin Immunol. 2021;148:627–632.e3. 33819506 10.1016/j.jaci.2021.03.028 PMC8355025

[75] Shah A , Kang S, Talley NJ, et al The duodenal mucosa associated microbiome, visceral sensory function, immune activation and psychological comorbidities in functional gastrointestinal disorders with and without self-reported non-celiac wheat sensitivity. Gut Microbes. 2022;14:2132078. 36303431 10.1080/19490976.2022.2132078 PMC9621048

[76] Cheng SH , Yang TY, Hsu CC, et al *Lactobacillus paragasseri* BBM171 ameliorates allergic airway inflammation induced by ovalbumin in mice via modulating the Th1/Th2 balance. Microorganisms. 2022;10:2041. 36296316 10.3390/microorganisms10102041 PMC9611844

[77] Chu DK , Jimenez-Saiz R, Verschoor CP, et al Indigenous enteric eosinophils control DCs to initiate a primary Th2 immune response in vivo. J Exp Med. 2014;211:1657–1672. 25071163 10.1084/jem.20131800 PMC4113937

[78] Abbas AK , Murphy KM, Sher A. Functional diversity of helper T lymphocytes. Nature. 1996;383:787–793. 8893001 10.1038/383787a0

[79] Skokos D , Nussenzweig MC. CD8- DCs induce IL-12-independent Th1 differentiation through Delta 4 Notch-like ligand in response to bacterial LPS. J Exp Med. 2007;204:1525–1531. 17576775 10.1084/jem.20062305 PMC2118646

[80] Vignali DA , Kuchroo VK. IL-12 family cytokines: immunological playmakers. Nat Immunol. 2012;13:722–728. 22814351 10.1038/ni.2366 PMC4158817

[81] Li N , Wang J, Liu P, et al Multi-omics reveals that Bifidobacterium breve M-16V may alleviate the immune dysregulation caused by nanopolystyrene. Environ Int. 2022;163:107191. 35325770 10.1016/j.envint.2022.107191

[82] Zhao Y , Qi C, Li X, et al Prevention of atopic dermatitis in mice by Lactobacillus reuteri Fn041 through induction of regulatory T cells and modulation of the gut microbiota. Mol Nutr Food Res. 2022;66:e2100699. 34825773 10.1002/mnfr.202100699

[83] Cho KH , Na HS, Jhun J, et al Lactobacillus (LA-1) and butyrate inhibit osteoarthritis by controlling autophagy and inflammatory cell death of chondrocytes. Front Immunol. 2022;13:930511. 36325344 10.3389/fimmu.2022.930511 PMC9619036

[84] Bui TPN , Manneras-Holm L, Puschmann R, et al Conversion of dietary inositol into propionate and acetate by commensal Anaerostipes associates with host health. Nat Commun. 2021;12:4798. 34376656 10.1038/s41467-021-25081-w PMC8355322

[85] Lu H , Xu X, Fu D, et al Butyrate-producing Eubacterium rectale suppresses lymphomagenesis by alleviating the TNF-induced TLR4/MyD88/NF-kappaB axis. Cell Host Microbe. 2022;30:1139–1150.e7. 35952646 10.1016/j.chom.2022.07.003

[86] Zhang Z , Yang P, Zhao J. Ferulic acid mediates prebiotic responses of cereal-derived arabinoxylans on host health. Anim Nutr. 2022;9:31–38. 35949987 10.1016/j.aninu.2021.08.004 PMC9344318

[87] Singh N , Gurav A, Sivaprakasam S, et al Activation of Gpr109a, receptor for niacin and the commensal metabolite butyrate, suppresses colonic inflammation and carcinogenesis. Immunity. 2014;40:128–139. 24412617 10.1016/j.immuni.2013.12.007 PMC4305274

[88] Ramiro-Puig E , Perez-Cano FJ, Castellote C, et al The bowel: a key component of the immune system. Rev Esp Enferm Dig. 2008;100:29–34. 18358058 10.4321/s1130-01082008000100006

[89] Gill PA , Muir JG, Gibson PR, et al A randomized dietary intervention to increase colonic and peripheral blood short-chain fatty acids modulates the blood B- and T-cell compartments in healthy humans. Am J Clin Nutr. 2022;116:1354–1367. 36084000 10.1093/ajcn/nqac246 PMC9630882

[90] Folkerts J , Redegeld F, Folkerts G, et al Butyrate inhibits human mast cell activation via epigenetic regulation of FcepsilonRI-mediated signaling. Allergy. 2020;75:1966–1978. 32112426 10.1111/all.14254 PMC7703657

