# Supplementary material for: Probiotics supplementation during pregnancy or infancy on multiple food allergies and gut microbiota: a systematic review and meta-analysis
Source: Nutr Rev. 2024 Mar 19;83(2):e25–41. doi: 10.1093/nutrit/nuae024 (PMC11723154; doi:10.1093/nutrit/nuae024)
Supplement: nuae024_Supplementary_Data [file nuae024_supplementary_data.docx]

# List of supplementary data

**Material S1:** Search strategy.

**Figure S1.** Non-linear dose-responses between (a) probiotic supplementation during pregnancy and infancy, (b) probiotic supplementation during infancy and the risk ratio in food allergy. (c) Non-linear dose-response between follow-up duration of probiotic supplementation during infancy and the risk ratio in food allergy. Shaded regions represented the 95% CI.

**Figure S2.** Forest plots of case-control studies for gut microbiota of alpha diversity in children with food allergy: diversity including (a) Shannon index and (b) Simpson index, (c) richness including Chao 1, (d) evenness including Observed OTUs.

**Table S1.** The measures of collection, preservation, extraction of intestinal samples and the methods of β-diversity analysis.

**Table S2.** Quality assessment of studies investigating probiotics supplementation during pregnancy and infancy on food allergy.

**Table S3.** Quality assessment of studies investigating perturbations of gut microbiota composition with food allergy in children.

**TableS4.** PRISMA 2020 checklist.

# Material S1. Search strategy.

**Search strategy for MEDLINE and EMBASE for probiotic supplementation on food allergy.**

(infant* OR infancy OR newborn* OR neonat* OR pediatric* OR paediatric* or toddler* or pre-schooler* or preschooler* or child or children or childhood or Pregnan* or maternal).ab,kw,ti.

AND

(probiotics or probiotic or Lactobacill* or Bifidobacteri* or lactococc* or Saccharomyces or (streptococcus and thermophilus) or (bacillus and subtilis) or (Enterococcus and faec*)).ab,kw,ti.

AND

(Food or nut or peanut or cashew or (tree nut) or (tree-nut) or egg or milk or shellfish or (shell fish) or shell-fish or wheat or almond).ab,kw,ti.

AND

(allerg* or hypersensiti* or hyper-sensiti* or sensiti*).ab,kw,ti.

**Search strategy for Pubmed and Cochrane Library for probiotic supplementation on food allergy.**

(infant* OR infancy OR newborn* OR neonat* OR pediatric* OR paediatric* or toddler* or pre-schooler* or preschooler* or child or children or childhood or Pregnan* or maternal)

AND

(probiotics or probiotic or Lactobacill* or Bifidobacteri* or lactococc* or Saccharomyces or (streptococcus and thermophilus) or (bacillus and subtilis) or (Enterococcus and faec*))

AND

(Food or nut or peanut or cashew or (tree nut) or (tree-nut) or egg or milk or shellfish or (shell fish) or shell-fish or wheat or almond)

AND

(allerg* or hypersensiti* or hyper-sensiti* or sensiti*)

**Search strategy for MEDLINE and EMBASE for perturbations of gut microbiota composition with food allergy.**

(gut or gastrointestinal or intestinal or feacal or fecal or stool).ab,kw,ti.

AND

(microbiome or microbiota or ecosystem or bacteria or flora or microflora or dysbiosis).ab,kw,ti.

AND

(Food or nut or peanut or cashew or (tree nut) or (tree-nut) or egg or milk or shellfish or (shell fish) or shell-fish or wheat or almond).ab,kw,ti.

AND

(allerg* or hypersensiti* or hyper-sensiti* or sensiti*).ab,kw,ti.

**Search strategy for Pubmed and Cochrane Library for perturbations of gut microbiota composition with food allergy.**

(gut or gastrointestinal or intestinal or feacal or fecal or stool)

AND

(microbiome or microbiota or ecosystem or bacteria or flora or microflora or dysbiosis)

AND

(Food or nut or peanut or cashew or (tree nut) or (tree-nut) or egg or milk or shellfish or (shell fish) or shell-fish or wheat or almond)

AND

(allerg* or hypersensiti* or hyper-sensiti* or sensiti*)


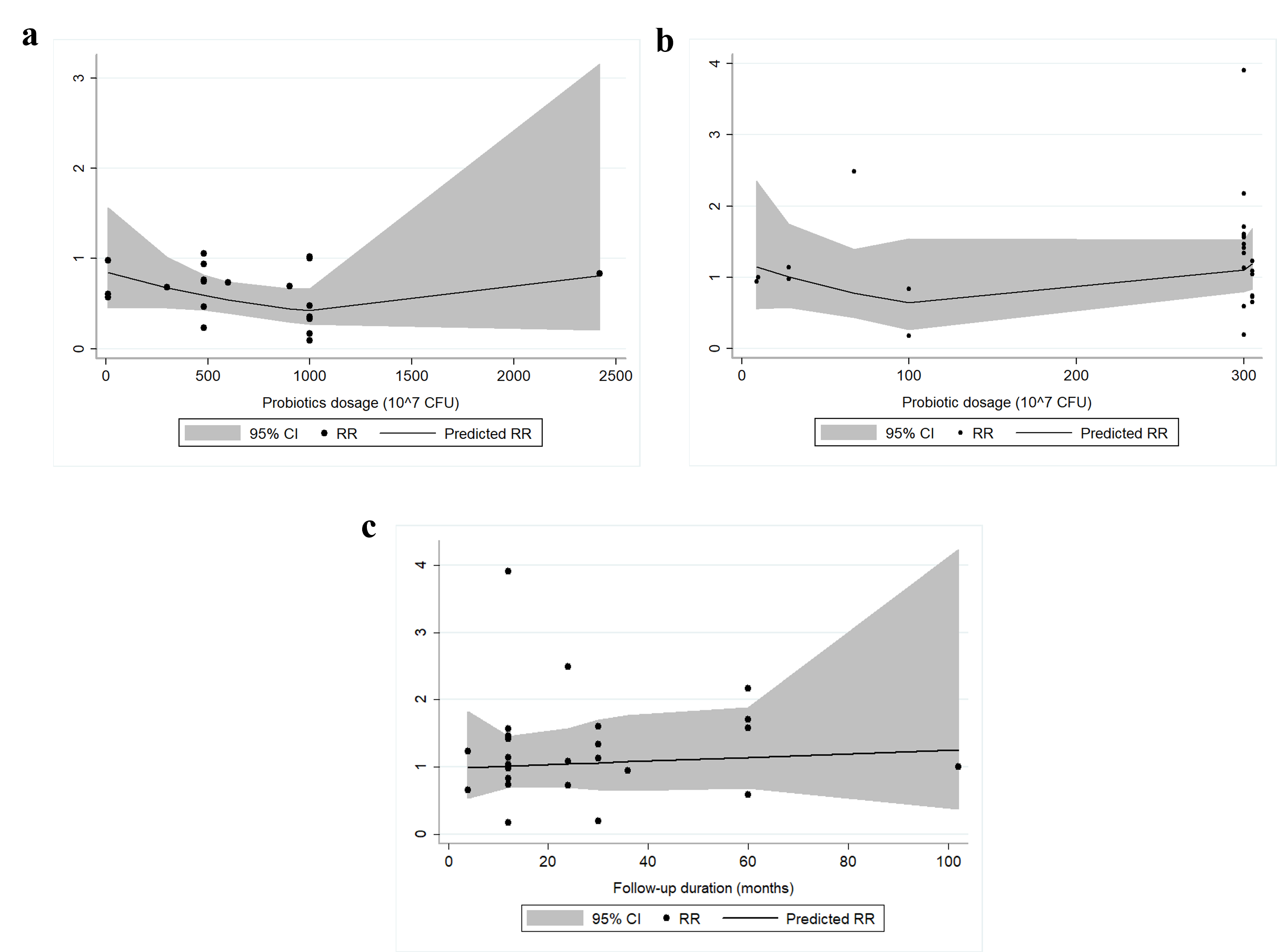


# Figure S1. Non-linear dose-responses between (a) probiotic supplementation during pregnancy and infancy, (b) probiotic supplementation during infancy and the risk ratio in food allergy. (c) Non-linear dose-response between follow-up duration of probiotic supplementation during infancy and the risk ratio in food allergy. Shaded regions represented the 95% CI. RR: Relative risk; CI: Confidence intervals; CFU: Colony-forming unit.


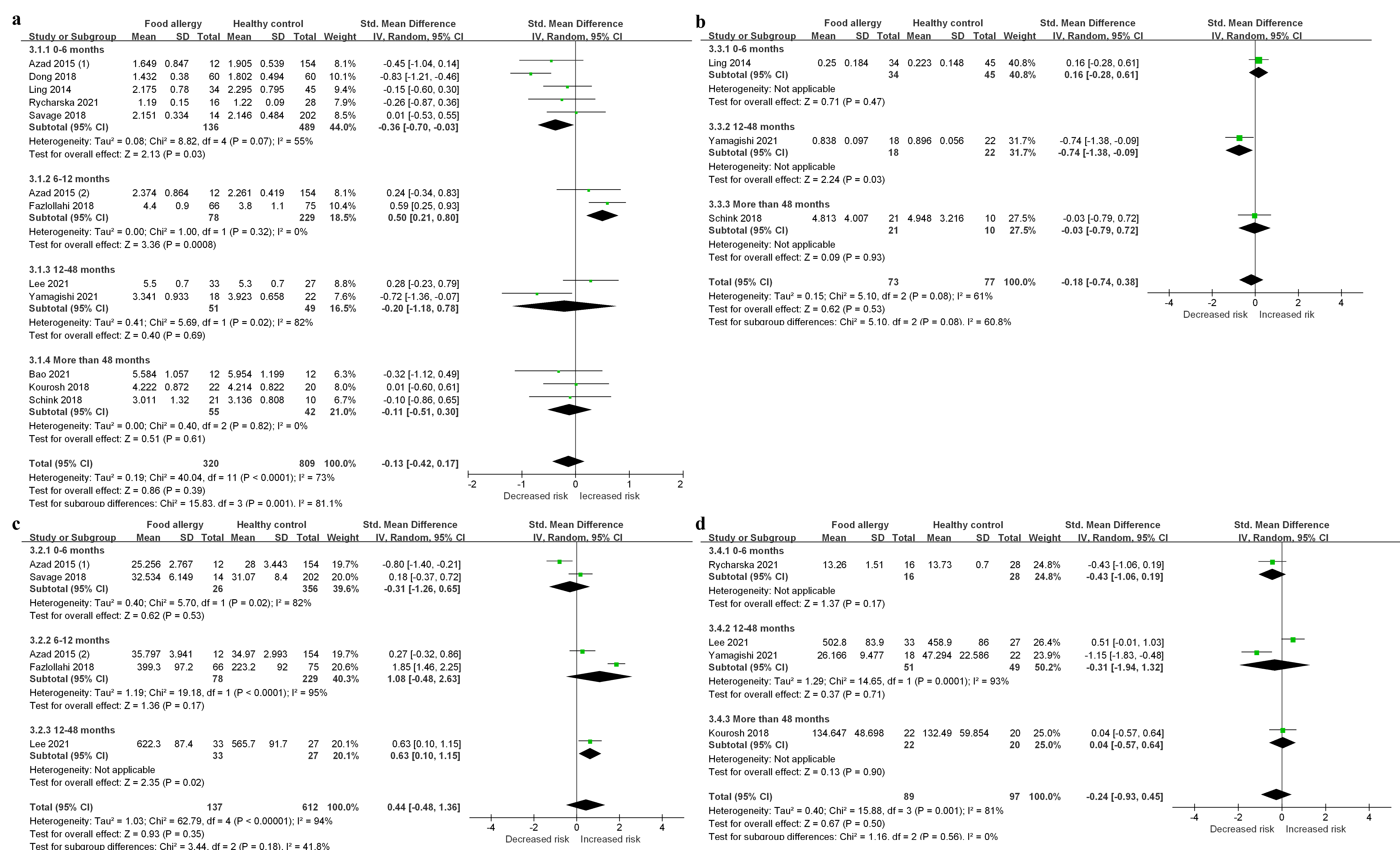


# Figure S2. Forest plots of case-control studies for gut microbiota of alpha diversity in children with food allergy: diversity including (a) Shannon index and (b) Simpson index, (c) richness including Chao 1, (d) evenness including Observed OTUs.

# Table S1. The measures of collection, preservation, extraction of intestinal samples and the methods of β-diversity analysis.

|  | | **Total** | | | | | **Beta diversity** | | |
| --- | --- | --- | --- | --- | --- | --- | --- | --- | --- |
| **study** | **Sequencing** | | **Diversity assessments** | **Collection & handling by participant** | **Long-term storage** | **DNA extraction method** | **Metric** | **Analysis** | **Finding** |
| Bao 2021 | 16S rRNA, qPCR | | α: Shannon; β: weighted UniFrac | - | - | Power Soil DNA Isolation Kit (MoBio) | weighted UniFrac | PERMANOVA | No significant difference |
| Bunyavanich 2016 | 16S rRNA V4 | | α: not given; β: unweighted UniFrac | - | - | MoBio Power Soil DNA Isolation kit (Carlsbad, CA) | unweighted UniFrac | PCoA,  PERMANOVA | 3-6months: significant difference;7-12months: no significant difference; 13-16months: no significant difference |
| Schink 2018 | 16S rRNA V3-V4 | | α: Shannon, Simpson; β: unweighted UniFrac | Stool samples were immediately cooled at 4ºC. | Stored within 4 hours at –20ºC till analysis | QIAamp Fast DNA Stool Mini Kit (Qiagen GmbH, Hilden, Germany) | unweighted UniFrac | PCoA,  NMDS | No significant difference |
| Du 2020 | 16S rRNA V3-V4 | | α: Chao1, Shannon; β: weighted and unweighted UniFrac | 2 g fresh fecal sample were put into 5 ml stool containers. Samples were frozen and transported immediately. | Stored at −80 °C until further analysis | PowerSoil DNA Isolation Kit (MoBio Laboratories, Carlsbad, CA, USA) | weighted UniFrac and unweighted UniFrac | PCoA | No significant difference |
| Fazlollahi 2018 | 16S rRNA V4, metagenome | | α: Chao1, Faith’s PD, Shannon; β: unweighted UniFrac | Stool collection kit provided by CoFAR was used. Stool samples were transported with ice packs. | Frozen at −80C upon arrival | MoBio Power Soil DNA Isolation kit (Carlsbad, CA) | unweighted UniFrac | PERMANOVA | Significant difference |
| Goldberg 2020 | 16S rRNA V4 | | α: Faith’s PD; β: weighted and unweighted UniFrac | - | Stored at − 80 °C until use | Mobio PowerSoil DNA extraction kit (MoBio, Carlsbad, CA) | weighted and unweighted UniFrac | PCoA | Significant difference |
| Kourosh 2018 | 16S rRNA V4 | | α: Shannon | - | - | MoBio Powersoil DNA kit (Mo Bio  Laboratories, Inc., Solana Beach, CA) | - | - | - |
| Lee 2021 | 16S rRNA V3-V4 | | α: Chao1, Observed OTUs, Shannon; β: weighted and unweighted UniFrac | Faecal collection kits were provided. samples were transported on ice within 2 hours. | Stored at − 80 °C | QIAamp DNA Stool Mini Kit (Qiagen, Germany) | weighted and unweighted UniFrac | PCoA, Adonis/  PERMANOVA | No significant difference |
| Savage 2018 | 16S rRNA V3-V5 | | α: Chao1, Shannon | Samples were store the sample in the freezer at home until bringing it. | - | - | - | **-** | **-** |
| Dong.P 2018 | 16S rRNA V3-V4 | | α: Shannon; β: unweighted UniFrac | Samples were refrigerated during transport. | Stored at − 80 °C | QIAamp DNA stool mini kit (Qiagen, Hilden, Germany) | unweighted UniFrac | PERMANOVA | Significant difference |
| Ling 2014 | 16S rRNA V1-V3 | | α: Shannon, Simpson, and evenness indices, ACE and Chao1; β: unweighted UniFrac | Samples were collected in a sterile plastic cup and was kept in an ice box. | Stored at −80°C after preparation within 15 min | QIAamp DNA stool minikit (Qiagen, Hilden, Germany) | unweighted UniFrac | PCoA | No significant difference |
| Rycharska 2021 | 16S rRNA V3-V4 | | α: Shannon, number of OTU, Shannon’s evenness; β: unweighted UniFrac | Sterile, disposable equipment was used throughout the sampling procedure. Samples were frozen for transport to the laboratory. | Stored at −80 °C | using bead-beating (on PowerLyzer, MoBio) combined with flocculation and silica columns | unweighted UniFrac | RDA | Significant difference |
| Yamagishi 2021 | 16S rRNA | | α: Shannon, Simpson; β: Bray-Curtis | - | - | - | Bray-Curtis | PCoA | Significant difference |
| Azad 2015 | 16S rRNA V4 | | α: Chao1, Shannon; β: unweighted UniFrac | Samples were refrigerated during transport. | Stored at -80°C | QIAamp DNA Stool Mini   Kit (Qiagen, Venlo, Netherlands). | unweighted UniFrac | PREMANOVA | Significant difference |
| Tanaka 2017 | 16S rRNA V1-V2, 16S rRNA and qPCR VI-V8 for Clostridium species | | α: number of detected OTUs, PD whole tree, Shannon; β: unweighted UniFrac | Samples were gathered into collection tubes and frozen for transport to the laboratory. | Stored at -80°C | Bead beating method | unweighted UniFrac | PLS-DA | Significant difference |
| Dong.Y 2018 | 16S rRNA V3-V4 | | α: Chao1, ACE, Simpson, Shannon, and coverage indices | Samples were transferred into sterile plastic tubes. | Stored at -80°C | TIANamp Stool DNA Kit (Tiangen, China, cat#DP328-02) | - | - | - |
| Guo 2016 | PCR V3 | | - | All feces samples were collected into sterile plastic tubes. | Stored at -80°C | QIAamp DNA Stool Mini Kit (Qiagen, Hilden, Germany) | - | - | - |

# Table S2. Quality assessment of studies investigating probiotics supplementation during pregnancy and infancy on food allergy.

| **Study** | **Random Sequence Generation** | **Allocation Concealment** | **Blinding of participants and personnel** | **Blinding of outcome assessment** | **Incomplete outcome data** | **Selective Reporting** | **Other bias** |
| --- | --- | --- | --- | --- | --- | --- | --- |
| Allen 2014 | Low | Low | Low | Low | Unclear | Low | Low |
| Kallio 2019 | Unclear | Unclear | Low | Low | Low | Low | Unclear |
| Abrahamsson 2007 | Low | Low | Low | Low | Low | Low | Low |
| Wickens 2008 | low | low | low | low | low | low | low |
| Niers 2009 | Unclear | Low | Low | Low | Low | High | High |
| Kim 2010 | Low | Unclear | Low | Low | High | High | High |
| Boyle 2011 | Low | Unclear | Low | Low | Low | Low | Low |
| Kalliomaki 2003 | Low | Low | Low | Low | Low | Unclear | Low |
| Plummer 2019 | Unclear | Low | Low | Low | Low | Low | Unclear |
| Morisset 2008 | Low | Low | Low | Low | Low | Low | Low |
| Taylor 2007 | Low | Low | Low | Low | Low | Low | Low |
| Prescott 2008 | Low | Low | Low | Low | Low | Low | Low |
| Jensen 2012 | Low | Low | Low | Low | Low | Low | Low |
| West 2013 | Unclear | Unclear | Low | Low | Low | Low | Unclear |
| Soh 2009 | Low | Low | Low | Low | Low | Low | Low |
| Rautava 2006 | Low | unclear | unclear | Low | High | unclear | unclear |
| Lau 2012 | Low | Low | Low | Low | Low | Low | Low |

# Table S3. Quality assessment of studies investigating perturbations of gut microbiota composition with food allergy in children.

|  | **Selection** | | | | **Comparability** | **Exposure** | | |  |
| --- | --- | --- | --- | --- | --- | --- | --- | --- | --- |
| **Study** | **Is the case definition adequate?** | **Representativeness of the cases** | **Selection of Controls** | **Definition of Controls** | **Comparability of cases and controls on the basis of the design or analysis** | **Ascertainment of exposure** | **Same method of ascertainment for cases and controls** | **Non-Response rate** | **Total** |
| Bao 2021 | ☆ | ☆ | - | ☆ | - | ☆ | ☆ | ☆ | ☆☆☆☆☆☆ |
| Bunyavanich 2016 | ☆ | ☆ | ☆ | ☆ | ☆ | ☆ | ☆ | ☆ | ☆☆☆☆☆☆☆☆ |
| Schink 2018 | - | ☆ | ☆ | - | - | ☆ | ☆ | - | ☆☆☆☆ |
| Du 2020 | ☆ | ☆ | - | ☆ | - | ☆ | ☆ | ☆ | ☆☆☆☆☆☆ |
| Fazlollahi 2018 | ☆ | ☆ | - | ☆ | ☆☆ | ☆ | ☆ | ☆ | ☆☆☆☆☆☆☆☆ |
| Goldberg 2020 | ☆ | ☆ | - | ☆ | - | ☆ | - | ☆ | ☆☆☆☆☆ |
| Kourosh 2018 | ☆ | ☆ | ☆ | ☆ | ☆☆ | ☆ | ☆ | ☆ | ☆☆☆☆☆☆☆☆☆ |
| Lee 2021 | ☆ | ☆ | ☆ | ☆ | - | ☆ | ☆ | ☆ | ☆☆☆☆☆☆☆ |
| Savage 2018 | ☆ | ☆ | ☆ | ☆ | - | ☆ | ☆ | ☆ | ☆☆☆☆☆☆☆ |
| Dong 2018 | ☆ | ☆ | - | ☆ | - | ☆ | ☆ | ☆ | ☆☆☆☆☆☆ |
| Ling 2014 | ☆ | ☆ | ☆ | ☆ | - | ☆ | ☆ | ☆ | ☆☆☆☆☆☆☆ |
| Rycharska 2021 | ☆ | ☆ | - | ☆ | - | ☆ | ☆ | ☆ | ☆☆☆☆☆☆ |
| Yamagishi 2021 | ☆ | - | - | ☆ | - | ☆ | ☆ | - | ☆☆☆☆ |
| Azad 2015 | ☆ | ☆ | ☆ | ☆ | ☆ | ☆ | ☆ | - | ☆☆☆☆☆☆☆ |
| Tanaka 2017 | ☆ | ☆ | ☆ | ☆ | - | ☆ | ☆ | - | ☆☆☆☆☆☆ |
| Dong 2018 | ☆ | ☆ | ☆ | ☆ | - | ☆ | ☆ | ☆ | ☆☆☆☆☆☆☆ |
| Guo 2016 | ☆ | ☆ | ☆ | ☆ | - | ☆ | ☆ | ☆ | ☆☆☆☆☆☆☆ |

☆ refers to 1 point.

# TableS4. PRISMA 2020 checklist

| **Section and Topic** | **Item #** | **Checklist item** | **Location where item is reported** |
| --- | --- | --- | --- |
| **TITLE** | | |  |
| Title | 1 | Identify the report as a systematic review. | Page 1 |
| **ABSTRACT** | | |  |
| Abstract | 2 | See the PRISMA 2020 for Abstracts checklist. | Page 3-4 |
| **INTRODUCTION** | | |  |
| Rationale | 3 | Describe the rationale for the review in the context of existing knowledge. | Page 5-6 |
| Objectives | 4 | Provide an explicit statement of the objective(s) or question(s) the review addresses. | Page 6 |
| **METHODS** | | |  |
| Eligibility criteria | 5 | Specify the inclusion and exclusion criteria for the review and how studies were grouped for the syntheses. | Page 7 |
| Information sources | 6 | Specify all databases, registers, websites, organisations, reference lists and other sources searched or consulted to identify studies. Specify the date when each source was last searched or consulted. | Page 7 |
| Search strategy | 7 | Present the full search strategies for all databases, registers and websites, including any filters and limits used. | Material S1 |
| Selection process | 8 | Specify the methods used to decide whether a study met the inclusion criteria of the review, including how many reviewers screened each record and each report retrieved, whether they worked independently, and if applicable, details of automation tools used in the process. | Page 7, Table 1 and Table 2 |
| Data collection process | 9 | Specify the methods used to collect data from reports, including how many reviewers collected data from each report, whether they worked independently, any processes for obtaining or confirming data from study investigators, and if applicable, details of automation tools used in the process. | Page 7 |
| Data items | 10a | List and define all outcomes for which data were sought. Specify whether all results that were compatible with each outcome domain in each study were sought (e.g. for all measures, time points, analyses), and if not, the methods used to decide which results to collect. | Page 7-8 |
|  | 10b | List and define all other variables for which data were sought (e.g. participant and intervention characteristics, funding sources). Describe any assumptions made about any missing or unclear information. | Page 7 |
| Study risk of bias assessment | 11 | Specify the methods used to assess risk of bias in the included studies, including details of the tool(s) used, how many reviewers assessed each study and whether they worked independently, and if applicable, details of automation tools used in the process. | Page 8 |
| Effect measures | 12 | Specify for each outcome the effect measure(s) (e.g. risk ratio, mean difference) used in the synthesis or presentation of results. | Page 8 |
| Synthesis methods | 13a | Describe the processes used to decide which studies were eligible for each synthesis (e.g. tabulating the study intervention characteristics and comparing against the planned groups for each synthesis (item #5)). | Page 8 |
|  | 13b | Describe any methods required to prepare the data for presentation or synthesis, such as handling of missing summary statistics, or data conversions. | Page 8 |
|  | 13c | Describe any methods used to tabulate or visually display results of individual studies and syntheses. | Page 8 |
|  | 13d | Describe any methods used to synthesize results and provide a rationale for the choice(s). If meta-analysis was performed, describe the model(s), method(s) to identify the presence and extent of statistical heterogeneity, and software package(s) used. | Page 8 |
|  | 13e | Describe any methods used to explore possible causes of heterogeneity among study results (e.g. subgroup analysis, meta-regression). | Page 8 |
|  | 13f | Describe any sensitivity analyses conducted to assess robustness of the synthesized results. | Page 8 |
| Reporting bias assessment | 14 | Describe any methods used to assess risk of bias due to missing results in a synthesis (arising from reporting biases). | Page 8 |
| Certainty assessment | 15 | Describe any methods used to assess certainty (or confidence) in the body of evidence for an outcome. | Page 8 |
| **RESULTS** | | |  |
| Study selection | 16a | Describe the results of the search and selection process, from the number of records identified in the search to the number of studies included in the review, ideally using a flow diagram. | Figure 1 |
|  | 16b | Cite studies that might appear to meet the inclusion criteria, but which were excluded, and explain why they were excluded. | Figure 1 |
| Study characteristics | 17 | Cite each included study and present its characteristics. | Page 9-10 |
| Risk of bias in studies | 18 | Present assessments of risk of bias for each included study. | Table S2-S3 |
| Results of individual studies | 19 | For all outcomes, present, for each study: (a) summary statistics for each group (where appropriate) and (b) an effect estimate and its precision (e.g. confidence/credible interval), ideally using structured tables or plots. | Figure 2-4, Figure S2 |
| Results of syntheses | 20a | For each synthesis, briefly summarise the characteristics and risk of bias among contributing studies. | Page 8-10 |
|  | 20b | Present results of all statistical syntheses conducted. If meta-analysis was done, present for each the summary estimate and its precision (e.g. confidence/credible interval) and measures of statistical heterogeneity. If comparing groups, describe the direction of the effect. | Page 8-14 |
|  | 20c | Present results of all investigations of possible causes of heterogeneity among study results. | Page 9-14 |
|  | 20d | Present results of all sensitivity analyses conducted to assess the robustness of the synthesized results. | Page 9-10 |
| Reporting biases | 21 | Present assessments of risk of bias due to missing results (arising from reporting biases) for each synthesis assessed. | Table S2-S3 |
| Certainty of evidence | 22 | Present assessments of certainty (or confidence) in the body of evidence for each outcome assessed. | Table S2-S3 |
| **DISCUSSION** | | |  |
| Discussion | 23a | Provide a general interpretation of the results in the context of other evidence. | Page 14-21 |
|  | 23b | Discuss any limitations of the evidence included in the review. | Page 20-21 |
|  | 23c | Discuss any limitations of the review processes used. | Page 20-21 |
|  | 23d | Discuss implications of the results for practice, policy, and future research. | Page 21 |
| **OTHER INFORMATION** | | |  |
| Registration and protocol | 24a | Provide registration information for the review, including register name and registration number, or state that the review was not registered. | Page 7 |
|  | 24b | Indicate where the review protocol can be accessed, or state that a protocol was not prepared. | Page 7 |
|  | 24c | Describe and explain any amendments to information provided at registration or in the protocol. | Not applicable |
| Support | 25 | Describe sources of financial or non-financial support for the review, and the role of the funders or sponsors in the review. | Page 21-22 |
| Competing interests | 26 | Declare any competing interests of review authors. | Page 22 |
| Availability of data, code and other materials | 27 | Report which of the following are publicly available and where they can be found: template data collection forms; data extracted from included studies; data used for all analyses; analytic code; any other materials used in the review. | Page 22-23, 38-39 |
